# Association between the difference in cystatin C and creatinine-based eGFR and risks of multiple cardiovascular diseases: a prospective cohort study

**DOI:** 10.3389/fmed.2025.1670059

**Published:** 2025-11-17

**Authors:** Zhiyu Qiao, Xinyi Liu, Hao Liu, Suwei Chen, Chengnan Li, Yipeng Ge, Haiou Hu, Junming Zhu

**Affiliations:** Department of Cardiovascular Surgery, Beijing Aortic Disease Center, Beijing Anzhen Hospital of Capital Medical University, Beijing, China

**Keywords:** estimated glomerular filtration rate, serum creatinine, cystatin C, cardiovascular disease, all-cause mortality

## Abstract

**Background:**

The difference between cystatin C- and creatinine-based estimated glomerular filtration rate (eGFR_diff_) is closely associated with various adverse outcomes. This study aims to comprehensively evaluate the association between eGFR_diff_, all-cause mortality, and the risk of multiple cardiovascular-related diseases.

**Methods:**

This study analyzed data from 297,140 participants in the UK Biobank to assess the association between eGFR_diff_, mortality, and the incidence of multiple cardiovascular-related diseases. eGFR_diff_ was classified into three groups: negative (< −15 mL/min/1.73 m^2^), intermediate (−15 to 15 mL/min/1.73 m^2^), and positive (≥ 15 mL/min/1.73 m^2^). Cox proportional hazards regression models were used to evaluate this association, while various sensitivity analyses were performed to assess its robustness.

**Results:**

During a mean follow-up of 13.1 years, the positive eGFR_diff_ group exhibited significantly lower mortality, cardiovascular disease (CVD) incidence, and the occurrence of CVD-related conditions. In the fully adjusted model, participants in the negative eGFR_diff_ group had a hazard ratio of 1.44 (95% confidence interval [CI], 1.40–1.49) for all-cause mortality, 1.49 (95% CI, 1.41–1.59) for CVD incidence, and 1.25 (95% CI, 1.22–1.27) for CVD mortality. The risk of all 10 CVD-related conditions was also significantly higher in the negative group, whereas the positive group exhibited significantly lower risks. For every 10 mL/min/1.73 m^2^ increase in eGFR_diff_, the incidence of various diseases decreased by approximately 10–19%.

**Conclusion:**

eGFR_diff_ is significantly associated with increased risks of mortality, CVD incidence, and multiple CVD-related conditions. These findings underscore the critical need for developing targeted prevention strategies, particularly for populations with reduced eGFR_diff_.

## Introduction

Chronic kidney disease is a global public health issue, affecting more than 10% of adults worldwide (1). Impaired kidney function is associated with a high risk of cardiovascular disease (CVD) and all-cause mortality (2). Numerous studies have suggested that non-dialysis-dependent kidney dysfunction is closely linked to heart failure, atrial fibrillation, and the incidence of CVD in asymptomatic populations (3, 4). Therefore, identifying high-risk individuals with kidney dysfunction and implementing early interventions are crucial for preventing the onset and progression of cardiovascular disease (5).

Estimating glomerular filtration rate (eGFR) using serum creatinine or cystatin C is a widely adopted method for assessing kidney function in clinical practice (6). However, in recent years, the substantial intra-individual differences between cystatin C-based eGFR (eGFR_cys_) and creatinine-based eGFR (eGFR_cr_) have been increasingly recognized and reported to be associated with various adverse outcomes, including mortality, end-stage kidney disease, and hospitalization (7, 8). These differences may be influenced by multiple non-renal factors, such as muscle mass, lifestyle, and chronic diseases (9, 10). The eGFR difference (eGFR_diff_), defined as eGFR_cys_ minus eGFR_cr_, has recently been proposed as a marker of overall health status (11). Previous studies have shown that a more negative eGFR_diff_ is associated with several adverse cardiovascular outcomes, including heart failure, atrial fibrillation, and atherosclerotic cardiovascular disease (12–14). Despite these associations, no large-scale cohort study has systematically analyzed the relationship between eGFR_diff_ and the incidence of comprehensive CVD as well as a wide range of CVD-related conditions.

This study aims to investigate the association between eGFR_diff_ and the incidence, mortality, and various CVD-related diseases using prospective data from the UK Biobank.

## Materials and methods

### Study design and participants

We drew upon data from the UK Biobank study (Application Number 145937), a comprehensive prospective cohort comprising more than 500,000 individuals aged 37–73 years, recruited from 22 assessment centers throughout the United Kingdom between 2006 and 2010. Participants contributed extensive health-related data via a touchscreen questionnaire, which encompassed demographics, socio-economic status, lifestyle habits, and medical conditions. The study’s methodology and data collection processes have been extensively documented in previous publications (15).

The cohort initially included 466,571 participants with complete eGFR data at baseline. After excluding individuals with a history of cardiovascular disease at baseline, as well as those with missing demographic information or other covariate data, the final analysis included 297,140 participants (Supplementary Figure 1).

### Main exposure

In this study, the primary exposure of interest was the non-race-based eGFR_diff_. It was calculated using baseline serum cystatin C and creatinine levels, applying the CKD-EPI (Chronic Kidney Disease Epidemiology Collaboration) equations. Specifically, eGFR_diff_ was derived by subtracting the non-race-based eGFR_cr_ calculated from creatinine from the eGFR_cys_ calculated from cystatin C. Participants were categorized into three groups based on eGFR_diff_ values: negative (< −15 mL/min/1.73 m^2^), intermediate (−15 to 15 mL/min/1.73 m^2^), and positive (≥ 15 mL/min/1.73 m^2^). Additionally, eGFR_diff_ was analyzed as a continuous variable per 10 mL/min/1.73 m^2^ increment. In a sensitivity analysis, we used the race-related eGFR_diff_ as the primary exposure, defined as eGFR_cys_ minus the race-related eGFR_cr_.

### Assessment of covariates

Covariates included demographic characteristics, baseline medical history, lifestyle factors, chronic inflammation markers, and laboratory biochemical test indicators. Demographic characteristics included age, sex, education level, self-reported race, townsend deprivation index, employment status, body mass index (BMI), handgrip strength (HGS) and appendicular skeletal muscle mass (ASM). HGS was measured in both hands in kilograms, and the average grip strength of both hands was calculated. Body composition was assessed at the baseline visit using a single-frequency segmental body composition analyzer. Muscle and fat mass were derived using bioelectrical impedance analysis. ASM was calculated as the sum of lean soft tissue mass in the upper and lower limbs. Finally, sarcopenia was identified based on the criteria established by the Foundation for the National Institutes of Health (FNIH) Sarcopenia Project. Baseline medical history also covered the presence of chronic respiratory disease, chronic liver disease, hypertension, diabetes, and dyslipidemia.

Lifestyle factors were evaluated using six components aligned with World Health Organization guidelines: dietary habits, sleep patterns (classified as healthy, moderate, or unhealthy), physical activity levels (high, moderate, or low), sedentary behavior (low, moderate, or high), and history of smoking and alcohol consumption. Comprehensive details regarding the assessment of these lifestyle factors can be found in Supplementary Tables 1, 2. For each lifestyle factor, a score of 0 was assigned to an unhealthy level and 1 to a healthy level (similarly, for sleep patterns, a score of 1 was assigned to a moderate sleep health pattern and 2 to the healthiest sleep pattern). The six lifestyle factors were then summed to generate a composite healthy lifestyle score.

To account for the role of chronic inflammation, we also analyzed various inflammatory markers, including white blood cell (WBC) count, platelet count, C-reactive protein (CRP) levels, neutrophil count, lymphocyte count, and the neutrophil-to-lymphocyte ratio (NLR). Additionally, we calculated the low-grade chronic inflammation score (INFLA score) as a comprehensive measure of individual inflammatory status. Based on previous studies, the INFLA score, which is closely associated with multiple diseases (16), integrates four inflammatory markers: CRP, WBC count, platelet count, and NLR. To compute the INFLA score, each inflammatory marker was log-transformed. Biomarker levels within the highest deciles (7th–10th) were assigned scores ranging from + 1 to + 4, whereas those within the lowest deciles (1st–4th) were assigned scores from −4 to −1 (17). The resulting INFLA score ranged from −16 to + 16, with higher scores indicating elevated levels of low-grade inflammation.

Finally, we included multiple laboratory biochemical test indicators, including serum albumin, high-density lipoprotein (HDL-C), low-density lipoprotein (LDL-C), triglycerides, and the urine albumin-to-creatinine ratio (UACR). The UACR was used to determine the presence of albuminuria in participants.

### Outcomes

The primary outcomes of this study included cardiovascular disease (CVD) incidence, CVD mortality, and all-cause mortality. Secondary outcomes encompassed all CVD-related components, including stroke, heart failure, atrial fibrillation, valvular heart disease, coronary atherosclerotic heart disease, aortic aneurysm, peripheral artery disease, deep vein thrombosis, pulmonary embolism, and arterial embolism. All disease diagnoses were determined based on death registries, primary care records, hospitalization data, and self-reported diagnoses. Outcomes were classified using ICD-9 and ICD-10 codes (International Classification of Diseases, Ninth and Tenth Revisions). The follow-up period extended from the baseline assessment (2006–2010) to the earliest occurrence of a relevant disease diagnosis, death, loss to follow-up, or the end of the follow-up period, whichever occurred first.

### Statistical analysis

The baseline characteristics of participants were presented as means with standard deviations (SD) for continuous variables and as proportions for categorical variables. Comparisons of continuous variables were performed using analysis of variance (ANOVA), while differences in categorical variables were assessed using the χ^2^-test.

Pearson correlation was used to examine the relationship between eGFR difference (eGFR_diff_) and clinical parameters. Cox proportional hazards regression models were applied to calculate the hazard ratios (HRs) and 95% confidence intervals (CIs) for eGFR_diff_ with respect to primary and secondary outcomes. Three multivariable-adjusted models were developed. Model 1 adjusted for age, sex, racial background, educational level, occupational status, Townsend deprivation index, and body mass index. Model 2 further adjusted for healthy lifestyle score and comorbidities (chronic respiratory disease, chronic liver disease, hypertension, diabetes, and dyslipidemia). Model 3 additionally adjusted for laboratory measurements (INFLA score, serum albumin, HDL-C, LDL-C, triglycerides, UACR, and eGFR_cr_). Restricted cubic splines (RCS) were used to explore the association between eGFR_diff_ and the risk of outcome events. In addition, subgroup analyses were performed to examine potential differences, stratifying participants by sex (male and female), age (≥ 60 and < 60 years), BMI (normal BMI: 18.5-24.9 and abnormal BMI), and the presence of comorbidities at baseline.

In this study, several sensitivity analyses were conducted to assess the robustness and consistency of the models. First, we used race-related eGFR difference as the primary exposure. Second, we adjusted for eGFR_cys_ or eGFR_cr–cys_ instead of eGFR_cr_. Third, the analysis was stratified by eGFR_cr_, eGFR_cys_, and eGFR_cr–cys_ categories: ≥ 90, 60–89, 45–59, and < 45 mL/min/1.73 m^2^. Fourth, eGFR_diff_ was further categorized into tertiles. Fifth, we further adjusted for participants with sarcopenia. Sixth, we adjusted for the components of lifestyle scores and the INFLA score rather than using the two scores as a whole. Seventh, individuals with comorbidities (chronic liver disease, chronic respiratory disease, hypertension, diabetes, dyslipidemia, and sarcopenia) were excluded to examine the impact of comorbidities on the outcomes. Eighth, events occurring within the first 3 years of follow-up were excluded to minimize potential bias from early events. Finally, multiple imputation was used to address missing covariate data and evaluate the impact of incomplete variables on the results.

All statistical analyses were performed using R software version 4.4.1 (R Foundation for Statistical Computing). *P*-values were two-sided, with a significance level set at *P* < 0.05.

## Results

### Baseline characteristics of participants

The baseline characteristics of the participants are presented in Table 1. This study included 297,140 individuals with a mean (SD) age of 58.0 (7.6) years. Among them, 135,954 (45.8%) were male, and 284,119 (95.6%) were White. The mean non-race-based eGFR difference (eGFR_diff_) was −0.5 ± 12.9 mL/min/1.73 m^2^. The distribution of race-based eGFR_diff_ is shown in Supplementary Table 3. Compared to participants with lower eGFR_diff_, those with higher eGFR_diff_ were more likely to be female, younger, more highly educated, have a healthier lifestyle, exhibit lower levels of chronic inflammation, and report a lower prevalence of other diseases. In the race-related eGFR_diff_ subgroup analysis, we similarly observed consistent findings.

**TABLE 1:** Baseline characteristics stratified by categories of non-race-based eGFR_diff_.

Characteristics	eGFR_diff_ Categories (mL/min/1.73 m^2^)			*P*-value
	Negative < −15 (*N* = 64,452)	Midrange−15 to 15 (*N* = 216,099)	Positive ≥ 15 (*N* = 16,589)	
**Demographics**				
Age (years)	58.0 ± 7.6	55.5 ± 8.1	52.6 ± 7.9	< 0.001
Male (%)	31936 (49.6%)	97096 (44.9%)	6922 (41.7%)	< 0.001
White ethnicity or race (%)	61686 (95.7%)	207450 (96.0%)	14983 (90.3%)	< 0.001
Townsend deprivation index	−1.2 ± 3.1	−1.7 ± 2.9	−1.6 ± 2.9	< 0.001
University or college degree (%)	19411 (30.1%)	81559 (37.7%)	6665 (40.2%)	< 0.001
Employed, student, or retired (%)	58620 (91.0%)	199296 (92.2%)	15247 (91.9%)	< 0.001
BMI	28.7 ± 5.1	26.6 ± 4.0	26.0 ± 3.6	< 0.001
Grip strength (kg)	30.2 ± 11.0	31.6 ± 10.9	33.4 ± 11.2	< 0.001
**Lifestyle**				
Physical activity (%)				< 0.001
Low	25001 (38.8%)	64948 (30.1%)	4212 (25.4%)	
Moderate	21342 (33.1%)	73411 (34.0%)	5479 (33.0%)	
High	18109 (28.1%)	77740 (36.0%)	6898 (41.6%)	
**Sleep patterns (%)**				< 0.001
Poor	19752 (30.6%)	77131 (35.7%)	6266 (37.8%)	
Moderate	40629 (63.0%)	129305 (59.8%)	9633 (58.1%)	
Good	4071 (6.3%)	9663 (4.5%)	690 (4.2%)	
No heavy alcohol (%)	35741 (55.5%)	97777 (45.2%)	7061 (42.6%)	< 0.001
Never smoking (%)	31848 (49.4%)	125757 (58.2%)	10452 (63.0%)	< 0.001
Healthy diet (%)	6442 (10.0%)	20155 (9.3%)	1252 (7.6%)	< 0.001
**Sedentary time (%)**				< 0.001
High	14426 (22.4%)	39560 (18.3%)	2964 (17.9%)	
Moderate	22454 (34.8%)	70666 (32.7%)	5137 (31.0%)	
Low	27572 (42.8%)	105873 (49.0%)	8488 (51.2%)	
Healthy lifestyle score	4.0 ± 1.5	4.2 ± 1.4	4.3 ± 1.4	< 0.001
**Medical history**				
Chronic respiratory diseases (%)	8863 (13.8%)	26294 (12.2%)	1938 (11.7%)	< 0.001
Chronic liver disease (%)	363 (0.6%)	441 (0.2%)	16 (0.1%)	< 0.001
Hypertension (%)	21763 (33.8%)	52357 (24.2%)	3181 (19.2%)	< 0.001
Hyperglycemia (%)	6325 (9.8%)	11308 (5.2%)	625 (3.8%)	< 0.001
Dyslipidemia (%)	33242 (51.6%)	77078 (35.7%)	4794 (28.9%)	< 0.001
**Inflammation**				
White blood cell count (×*10*∧9/L)	7.1 ± 1.7	6.6 ± 1.6	6.5 ± 1.6	< 0.001
Platelet count (×*10*∧9/L)	255 ± 58.9	251 ± 55.3	247 ± 54.1	< 0.001
Lymphocyte count (×*10*∧9/L)	2.0 ± 0.6	1.9 ± 0.6	1.9 ± 0.5	< 0.001
Neutrophil count (×*10*∧9/L)	4.3 ± 1.3	4.0 ± 1.2	3.9 ± 1.3	< 0.001
C-reactive protein (mg/L)	2.4 ± 1.9	1.6 ± 1.6	1.4 ± 1.4	< 0.001
NLR	2.3 ± 1.1	2.3 ± 1.1	2.3 ± 1.0	< 0.001
INFLA score	1.4 ± 5.9	−0.7 ± 5.9	−1.5 ± 6.0	< 0.001
**Biochemical detection**				
Albumin (g/L)	45.0 ± 2.6	45.5 ± 2.5	45.6 ± 2.6	< 0.001
High-density lipoprotein (mmol/L)	1.4 ± 0.4	1.5 ± 0.4	1.6 ± 0.4	< 0.001
Low-density lipoprotein (mmol/L)	3.7 ± 0.9	3.6 ± 0.8	3.5 ± 0.8	< 0.001
Triglycerides (mmol/L)	2.0 ± 1.1	1.6 ± 0.9	1.5 ± 0.9	< 0.001
**UACR (mg/g) (%)**				< 0.001
<30	60237 (93.5%)	203645 (94.2%)	16046 (96.7%)	
30–300	3970 (6.2%)	11954 (5.5%)	523 (3.2%)	
> 300	245 (0.4%)	500 (0.2%)	20 (0.1%)	
eGFR_cys_ (mL/min/1.73 m^2^)	75.4 ± 10.7	93.7 ± 13.6	103 ± 10.7	< 0.001
eGFR_cr_ (mL/min/1.73 m^2^)	98.0 ± 9.4	95.7 ± 12.5	80.3 ± 11.5	< 0.001
eGFR_diff_ (mL/min/1.73 m^2^)	−22.6 ± 6.3	−2.0 ± 7.4	22.3 ± 6.9	< 0.001

*P*-values were determined using the ANOVA test for continuous variables and the chi-square test for categorical variables. Abbreviations: eGFR_diff_, the difference between cystatin C–based estimated glomerular filtration rate and creatinine-based estimated glomerular filtration rate; BMI, Body mass index; NLR, Neutrophil-to-Lymphocyte Ratio; INFLA score, Low-grade chronic inflammation score; UACR, Urinary albumin-creatinine ratio.

In the correlation analysis, we identified significant associations between non-race-based eGFR_diff_ and multiple factors (Supplementary Table 4). Specifically, eGFR_diff_ exhibited significant negative correlations with age (γ = −0.19; *P* < 0.001), BMI (γ = −0.22; *P* < 0.001), history of alcohol consumption (γ = −0.10; *P* < 0.001), dyslipidemia (γ = −0.16; *P* < 0.001), and the INFLA score for chronic inflammation (γ = −0.17; *P* < 0.001). In contrast, significant positive correlations were observed with physical activity (γ = 0.11; *P* < 0.001), healthy lifestyle score (γ = 0.06; *P* < 0.001), and HDL-C levels (γ = 0.17; *P* < 0.001). Similar patterns of association were also observed in the race-related eGFR_diff_ subgroup analysis (Supplementary Table 5).

### Association between eGFR_diff_ and mortality and incident CVD

During a mean follow-up period of 13.1 years, the incidence of cardiovascular disease (CVD), CVD-related mortality, and all-cause mortality were 43,315 (14.6%), 4,634 (1.6%), and 19,289 (6.5%), respectively. As shown in Table 2, compared to the negative group of non-race-based eGFR_diff_, both the moderate and positive groups exhibited significantly lower rates of mortality and CVD incidence. Specifically, the CVD incidence in the positive group (6.36 per 1,000 person-years) was significantly lower than that in the negative group (15.51 per 1,000 person-years). A similar trend was observed across various CVD-related conditions, with the most notable finding being the significantly lower incidence of coronary atherosclerotic heart disease in the positive group (2.63 per 1,000 person-years) compared to the negative group (6.52 per 1,000 person-years). As presented in Supplementary Table 6, a similar trend was observed in the groups stratified by race-related eGFR_diff_.

**TABLE 2:** Incidence of cardiovascular disease and its components across three categories of non-race-based eGFR_diff_ levels.

Outcomes	Total (*N* = 297,140)	eGFR_diff_ Categories (mL/min/1.73 m^2^)		
		Negative < −15 (*N* = 64,452)	Midrange −15 to 15 (*N* = 216099)	Positive ≥ 15 (*N* = 16,589)
Incident CVD	43315 (14.6%)	13338 (20.7%)	28461 (13.2%)	1516 (9.1%)
Incidence rate^#^	10.48 (10.38–10.57)	15.51 (15.25–15.77)	9.37 (9.27–9.48)	6.36 (6.05–6.69)
CVD mortality	4634 (1.6%)	1771 (2.7%)	2758 (1.3%)	105 (0.6%)
Incidence rate	1.03 (1.00–1.06)	1.83 (1.74–1.91)	0.84 (0.81–0.88)	0.42 (0.35–0.51)
All-cause mortality	19289 (6.5%)	6652 (10.3%)	12088 (5.6%)	549 (3.3%)
Incidence rate	4.39 (4.33–4.46)	7.09 (6.93–7.27)	3.77 (3.71–3.84)	2.22 (2.04–2.41)
**Incidence of CVD components**				
Stroke	5762 (1.9%)	1805 (2.8%)	3772 (1.7%)	185 (1.1%)
Incidence rate	1.32 (1.29–1.36)	1.94 (1.86–2.04)	1.18 (1.15–1.22)	0.75 (0.65–0.86)
HF	6451 (2.2%)	2538 (3.9%)	3760 (1.7%)	153 (0.9%)
Incidence rate	1.48 (1.44–1.52)	2.74 (2.63–2.84)	1.18 (1.14–1.22)	0.62 (0.53–0.73)
AF	15990 (5.4%)	5150 (7.9%)	10341 (4.8%)	499 (3.0%)
Incidence rate	3.72 (3.66–3.78)	5.67 (5.51–5.82)	3.29 (3.23–3.35)	2.04 (1.87–2.23)
VHD	8711 (2.9%)	2765 (4.3%)	5672 (2.6%)	274 (1.7%)
Incidence rate	2.00 (1.96–2.05)	2.99 (2.88–3.11)	1.79 (1.74–1.83)	1.11 (0.99–1.25)
CAD	18601 (6.3%)	5873 (9.1%)	12089 (5.6%)	639 (3.9%)
Incidence rate	4.35 (4.29–4.42)	6.52 (6.36–6.69)	3.87 (3.79–3.93)	2.63 (2.43–2.84)
AA	1799 (0.6%)	622 (0.9%)	1127 (0.5%)	50 (0.3%)
Incidence rate	0.41 (0.39–0.43)	0.67 (0.62–0.72)	0.35 (0.33–0.37)	0.20 (0.15–0.27)
PAD	1871 (0.6%)	751 (1.2%)	1080 (0.5%)	40 (0.2%)
Incidence rate	0.43 (0.41–0.45)	0.76 (0.70–0.81)	0.34 (0.32–0.36)	0.16 (0.12–0.22)
DVT	2269 (0.8%)	706 (1.1%)	1449 (0.7%)	114 (0.7%)
Incidence rate	0.52 (0.49–0.54)	0.76 (0.70–0.81)	0.45 (0.43–0.48)	0.46 (0.38–0.55)
PE	4453 (1.5%)	1400 (2.2%)	2878 (1.3%)	175 (1.1%)
Incidence rate	1.02 (0.99–1.05)	1.50 (1.43–1.58)	0.90 (0.87–0.94)	0.71 (0.61–0.82)
AE	578 (0.2%)	206 (0.3%)	363 (0.2%)	9 (0.1%)
Incidence rate	0.13 (0.12–0.14)	0.22 (0.19–0.25)	0.11 (0.10–0.13)	0.04 (0.02–0.07)

^#^The incidence rates of the corresponding diseases per 1,000 person-years were calculated. eGFR_diff_, the difference between cystatin C–based estimated glomerular filtration rate and creatinine-based estimated glomerular filtration rate; CVD, Cardiovascular disease; HF, Heart failure; AF, Atrial fibrillation; VHD, Valvular heart disease; CAD, Coronary atherosclerotic heart disease; AA, Aortic aneurysm; PAD, Peripheral artery disease; DVT, Deep vein thrombosis; PE, Pulmonary embolism; AE, Arterial embolism.

Table 3 presents the association between non-race-based eGFR_diff_ and mortality and CVD incidence. In the fully adjusted model, both the negative and positive eGFR_diff_ groups were associated with the primary outcomes compared to the moderate eGFR_diff_ group. Specifically, individuals in the negative eGFR_diff_ group had a higher risk of CVD incidence (HR = 1.22, 95% CI: 1.19–1.25), CVD mortality (HR = 1.47, 95% CI: 1.37–1.56), and all-cause mortality (HR = 1.41, 95% CI: 1.36–1.46). Conversely, individuals in the positive eGFR_diff_ group exhibited a lower risk of CVD incidence (HR = 0.86, 95% CI: 0.81–0.90), CVD mortality (HR = 0.67, 95% CI: 0.55–0.81), and all-cause mortality (HR = 0.77, 95% CI: 0.70–0.84). When analyzed as a continuous variable, each 10 mL/min/1.73 m^2^ increase in eGFR_diff_ was associated with a 10–19% reduction in the risk of mortality and CVD incidence. A similar trend was observed in Supplementary Table 7, which presents the association between race-related eGFR_diff_ and these outcomes.

**TABLE 3:** Cardiovascular disease risk and mortality stratified by non-race-based eGFR_diff_ levels.

Non-race-based eGFRdiff	Model 1		Model 2		Model 3	
	HR (95% CI)	*P*-value	HR (95% CI)	*P*-value	HR (95% CI)	*P*-value
**Incident CVD**						
Negative < −15	1.25 (1.22–1.27)	< 0.001	1.23 (1.20–1.26)	< 0.001	1.22 (1.19–1.25)	< 0.001
Midrange −15 to 15	1 (Reference)		1 (Reference)		1 (Reference)	
Positive ≥ 15	0.89 (0.84–0.93)	< 0.001	0.89 (0.85–0.94)	< 0.001	0.86 (0.81–0.90)	< 0.001
Per 10 mL/min/1.73 m^2^ increase	0.90 (0.89–0.91)	< 0.001	0.91 (0.90–0.91)	< 0.001	0.90 (0.89–0.91)	< 0.001
**CVD mortality**						
Negative < −15	1.49 (1.41–1.59)	< 0.001	1.47 (1.38–1.57)	< 0.001	1.47 (1.37–1.56)	< 0.001
Midrange −15 to 15	1 (Reference)		1 (Reference)		1 (Reference)	
Positive ≥ 15	0.72 (0.59–0.87)	< 0.001	0.73 (0.59–0.88)	< 0.001	0.67 (0.55–0.81)	0.005
Per 10 mL/min/1.73 m^2^ increase	0.81 (0.79–0.83)	< 0.001	0.82 (0.80–0.84)	< 0.001	0.81 (0.79–0.83)	< 0.001
**All-cause mortality**						
Negative < −15	1.44 (1.40–1.49)	< 0.001	1.43 (1.38–1.47)	< 0.001	1.41 (1.36–1.46)	< 0.001
Midrange −15 to 15	1 (Reference)		1 (Reference)		1 (Reference)	
Positive ≥ 15	0.79 (0.73–0.87)	< 0.001	0.81 (0.74–0.88)	< 0.001	0.77 (0.70–0.84)	< 0.001
Per 10 mL/min/1.73 m^2^ increase	0.84 (0.83–0.85)	< 0.001	0.85 (0.84–0.86)	< 0.001	0.84 (0.83–0.85)	< 0.001

Model 1: Adjusted for age, sex, racial background, educational level, occupational status, Townsend deprivation index, and body mass index. Model 2: Further adjusted for healthy lifestyle score and comorbidities (chronic respiratory disease, chronic liver disease, hypertension, diabetes, and dyslipidemia). Model 3: Additionally adjusted for laboratory measurements (INFLA score, serum albumin, HDL-C, LDL-C, triglycerides, UACR, and eGFR_cr_). eGFR_diff_, the difference between cystatin C–based estimated glomerular filtration rate and creatinine-based estimated glomerular filtration rate; CVD, Cardiovascular disease; HR, hazard ratio; CI, confidence interval; INFLA score, Low-grade chronic inflammation score; HDL-C, high-density lipoprotein cholesterol; LDL-C, low-density lipoprotein cholesterol; UACR, Urinary albumin-creatinine ratio; eGFR_cr_, creatinine-based estimated glomerular filtration rate.

Table 4 and Supplementary Table 8 present the associations between both non-race-based and race-related eGFR_diff_ and the incidence of cardiovascular-related diseases. Compared to the moderate eGFR_diff_ group, individuals in the negative eGFR_diff_ group exhibited a significantly higher risk of multiple cardiovascular diseases, whereas those in the positive eGFR_diff_ group had a significantly lower risk. Among these, heart failure showed the most pronounced association, with an incidence risk ratio of 1.54 (95% CI: 1.46–1.62) in the negative group and 0.71 (95% CI: 0.60–0.84) in the positive group. Notably, among the 10 cardiovascular-related diseases analyzed, aortic aneurysm and deep vein thrombosis did not show significant differences in incidence in the positive eGFR_diff_ group, while significant associations were observed in the negative group. However, when eGFR_diff_ was treated as a continuous variable, significant associations were observed across all cardiovascular-related diseases, with each 10 mL/min/1.73 m^2^ increase in eGFR_diff_ corresponding to a 9–23% reduction in disease incidence.

**TABLE 4:** Risk of cardiovascular disease subtypes stratified by non-race-based eGFR_diff_ levels.

Characteristics	Negative < −15		Midrange −15 to 15	Positive ≥ 15		Per 10 mL/min/1.73 m^2^ increase	
	HR (95% CI)	*P*-value	HR (95% CI)	HR (95% CI)	*P*-value	HR (95% CI)	*P*-value
Stroke	1.25 (1.17–1.33)	< 0.001	1 (Reference)	0.81 (0.70–0.95)	0.008	0.89 (0.87–0.91)	< 0.001
HF	1.54 (1.46–1.62)	<0.001	1 (Reference)	0.71 (0.60–0.84)	<0.001	0.80 (0.78–0.82)	<0.001
AF	1.26 (1.21–1.31)	< 0.001	1 (Reference)	0.86 (0.78–0.94)	< 0.001	0.89 (0.88–0.90)	< 0.001
VHD	1.24 (1.18–1.30)	< 0.001	1 (Reference)	0.82 (0.73–0.93)	0.002	0.89 (0.87–0.91)	< 0.001
CAD	1.19 (1.15–1.23)	< 0.001	1 (Reference)	0.86 (0.80–0.94)	< 0.001	0.91 (0.90–0.93)	< 0.001
AA	1.32 (1.19–1.46)	<0.001	1 (Reference)	0.85 (0.63–1.13)	0.261	0.84 (0.81–0.88)	<0.001
PAD	1.55 (1.40–1.72)	< 0.001	1 (Reference)	0.68 (0.49–0.94)	0.018	0.81 (0.78–0.84)	< 0.001
DVT	1.35 (1.23–1.49)	< 0.001	1 (Reference)	1.02 (0.84–1.25)	0.834	0.90 (0.86–0.93)	< 0.001
PE	1.28 (1.19–1.37)	<0.001	1 (Reference)	0.85 (0.72–0.99)	0.043	0.89 (0.87–0.91)	<0.001
AE	1.35 (1.13–1.63)	< 0.001	1 (Reference)	0.42 (0.21–0.82)	0.011	0.83 (0.77—-0.89)	< 0.001

This analysis adjusted for age, sex, racial background, educational level, occupational status, Townsend deprivation index, body mass index, healthy lifestyle score, comorbidities (chronic respiratory disease, chronic liver disease, hypertension, diabetes, and dyslipidemia), and laboratory measurements (INFLA score, serum albumin, HDL-C, LDL-C, triglycerides, UACR, and eGFR_cr_). eGFR_diff_, the difference between cystatin C–based estimated glomerular filtration rate and creatinine-based estimated glomerular filtration rate; HR, hazard ratio; CI, confidence interval; INFLA score, Low-grade chronic inflammation score; HDL-C, high-density lipoprotein cholesterol; LDL-C, low-density lipoprotein cholesterol; UACR, Urinary albumin-creatinine ratio; eGFR_cr_, creatinine-based estimated glomerular filtration rate; HF, Heart failure; AF, Atrial fibrillation; VHD, Valvular heart disease; CAD, Coronary atherosclerotic heart disease; AA, Aortic aneurysm; PAD, Peripheral artery disease; DVT, Deep vein thrombosis; PE, Pulmonary embolism; AE, Arterial embolism.

**FIGURE 1:**
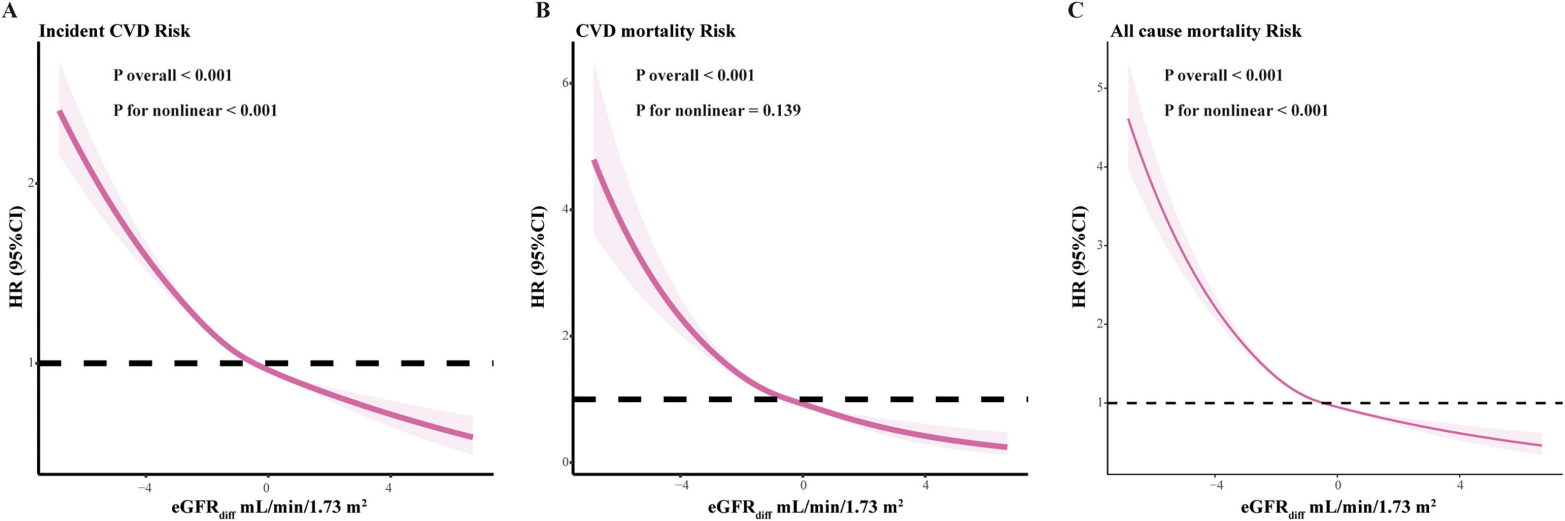
Restricted cubic spline plots of mortality and cardiovascular disease incidence based on non-race-based eGFR_diff_. This analysis adjusted for age, sex, racial background, educational level, occupational status, Townsend deprivation index, body mass index, healthy lifestyle score, comorbidities (chronic respiratory disease, chronic liver disease, hypertension, diabetes, and dyslipidemia), and laboratory measurements (eGFR_cr_, INFLA score, serum albumin, HDL-C, LDL-C, triglycerides, and UACR). eGFR_diff_, the difference between cystatin C–based estimated glomerular filtration rate and creatinine-based estimated glomerular filtration rate; HR, hazard ratio; CI, confidence interval; INFLA score, Low-grade chronic inflammation score; HDL-C, high-density lipoprotein cholesterol; LDL-C, low-density lipoprotein cholesterol; UACR, Urinary albumin-creatinine ratio; CVD, Cardiovascular disease.

Given the strong associations between eGFR_diff_ as a continuous variable and various diseases, we further explored its linear relationship using restricted cubic spline analysis. As shown in Figure 1 and Supplementary Figure 2, non-race-based eGFR_diff_ exhibited a significant linear association with CVD incidence, all-cause mortality, and several conditions including stroke, heart failure, and atrial fibrillation. However, no significant linear association was observed with CVD mortality. Similarly, as illustrated in Supplementary Figure 3, race-related eGFR_diff_ demonstrated comparable associations.

### Sensitivity analysis and subgroup analysis

To validate the robustness of the results, we conducted various sensitivity analyses. The associations between both types of eGFR_diff_ and mortality, CVD incidence, and cardiovascular-related disease incidence remained significant after adjusting for eGFRcys or eGFR_cr–cys_ instead of eGFR_cr_ (Supplementary Tables 9, 10) and when stratifying participants based on renal function using eGFR_cr_, eGFR_cys_, or eGFR_cr–cys_ (Supplementary Tables 11, 12). These associations persisted even in additional sensitivity analyses, including using tertiles of eGFR_diff_ and excluding participants who experienced relevant events within the first 3 years of follow-up (Supplementary Tables 13, 14).

Finally, we examined the effects of both types of eGFR_diff_ on mortality and cardiovascular disease incidence across various subgroups (Supplementary Tables 15–17). In all subgroups, eGFR_diff_ remained significantly associated with mortality and cardiovascular disease incidence, and no significant interactions were observed.

## Discussion

This study is a large prospective cohort analysis utilizing data from the UK Biobank to examine the association between eGFR_diff_ and mortality, as well as the incidence of CVD and its related conditions. Compared to participants with a positive eGFR_diff_, those with a negative eGFR_diff_ exhibited significantly higher mortality rates, CVD incidence, and the occurrence of 10 CVD-related diseases. Moreover, when eGFR_diff_ was treated as a continuous variable, higher eGFR_diff_ values were associated with significantly lower mortality and CVD incidence, independent of kidney function levels. These trends remained consistent across various sensitivity analyses and restricted cubic spline analyses.

Previous studies have reported associations between eGFR_diff_ and several adverse cardiovascular events. For instance, Debbie et al. observed in a cohort of 4,512 patients with chronic kidney disease that a negative baseline eGFR_diff_ was associated with an increased risk of heart failure (12). Similarly, Ga et al. reported a strong correlation between eGFR_diff_ and the incidence of atrial fibrillation (13). Furthermore, in a cohort of 9,092 participants from the Systolic Blood Pressure Intervention Trial (SPRINT), eGFR_diff_ was found to be closely associated with mortality risk (18). However, most of these studies focused on single cardiovascular outcomes or were conducted in relatively small cohorts. In this study, we emphasize that eGFR_diff_ is significantly associated with various CVD-related adverse events, as well as overall CVD incidence and mortality. Our findings provide additional evidence supporting the relationship between eGFR_diff_ and the occurrence of CVD and its related diseases. Interestingly, these associations remained significant even after adjusting for eGFR_cr_, eGFR_cys_, eGFR_cr–cys_, or stratifying by kidney function categories. This further reinforces the notion that a more negative eGFR_diff_ is a substantial risk factor for both mortality and CVD incidence. Notably, compared to the intermediate eGFR_diff_ group, we observed an increased risk of aortic aneurysm and deep vein thrombosis in the negative eGFR_diff_ group, similar to the elevated risks observed for other CVD conditions. However, this association was not significant in the positive eGFR_diff_ group. This discrepancy may be due to the relatively low number of cases for these two conditions within the cohort. Further large-scale cohort studies are needed to confirm this association.

Due to the complexity of eGFR measurement across different populations, efforts have been made in recent years to eliminate race-based kidney function assessment (19). In this context (20), the CKD-EPI equations have been updated to provide race-independent estimates for both eGFR_cr_ and eGFR_cys_. Consequently, variations in eGFR_diff_ have emerged due to differences in calculation methods. The UK Biobank includes participants with a diverse self-reported racial background. In this study, we systematically analyzed two classifications of eGFR_diff_ and found that both were significantly associated with mortality and CVD incidence. This finding has important implications for the prevention of CVD, particularly among traditionally understudied or high-risk racial groups.

Several potential mechanisms may underlie the association between eGFR_diff_ and the development of CVD. First, both creatinine and cystatin C are influenced by non-renal factors. Physical activity, chronic diseases, and muscle mass are key determinants of creatinine levels, while obesity, smoking, and steroid use are considered major non-renal factors affecting cystatin C levels (9, 10, 21). These influences contribute to the discrepancy between eGFR_cys_ and eGFR_cr_. A more negative eGFR_diff_ indicates a lower eGFR_cys_ and a higher eGFR_cr_, which may reflect poorer overall health status. Second, Grubb et al. hypothesized that individuals with a lower eGFR_diff_ tend to have higher BMI and elevated levels of inflammatory markers, a condition referred to as “shrunken pore syndrome” (22). Proteomic studies have shown that certain inflammatory proteins, such as interleukin-6 and osteoprotegerin, accumulate in individuals with shrunken pore syndrome (23). These inflammatory mediators are believed to contribute to endothelial damage, promote inflammation, and accelerate atherosclerosis—key pathogenic factors in CVD development (24). In our study, correlation analyses between eGFR_diff_ and various clinical indicators revealed that eGFR_diff_ was significantly positively associated with overall healthy lifestyle factors and negatively associated with chronic inflammation levels and preexisting disease history. These findings further support the proposed mechanisms linking eGFR_diff_ with CVD risk.

Our study has several notable strengths. First, it is a large-scale prospective cohort study that provides comprehensive and detailed data on the association between eGFR_diff_, mortality, and cardiovascular disease incidence. Second, this study systematically analyzes the relationship between eGFR_diff_ and 10 CVD-related conditions, addressing the limitations of previous research that primarily focused on single CVD outcomes. Additionally, we incorporated a wide range of potential confounders and constructed lifestyle and chronic inflammation scores, which enhance the robustness and generalizability of our findings.

However, our study has several limitations. First, as an observational study, it cannot establish a causal relationship between eGFR_diff_, mortality, and CVD development. Second, we were unable to fully adjust for potential residual confounders, such as the impact of unmeasured comorbidities. Third, eGFR_diff_ in this study was calculated based on baseline measurements, and we could not assess its changes over time. Fourth, because cystatin C is not routinely measured in all clinical settings, its applicability and generalizability may be limited. Finally, since the UK Biobank primarily consists of a predominantly White adult population, the generalizability of our findings to other racial and ethnic groups remains limited.

## Conclusion

In conclusion, this study found that eGFR_diff_ is closely associated with mortality, CVD incidence, and the risk of multiple CVD-related conditions. This finding underscores the importance of developing targeted prevention strategies, particularly for individuals with lower eGFR_diff_. However, further comprehensive evaluations are needed to determine the clinical utility of eGFR_diff_ as a predictive marker in medical practice.

## Data Availability

The original contributions presented in this study are included in the article/Supplementary material, further inquiries can be directed to the corresponding authors.
