# Supplementary material for: Association between the difference in cystatin C and creatinine-based eGFR and risks of multiple cardiovascular diseases: a prospective cohort study
Source: Front Med (Lausanne). 2025 Nov 17;12:1670059. doi: 10.3389/fmed.2025.1670059 (PMC12665693; doi:10.3389/fmed.2025.1670059)
Supplement: Supplementary file 1 [file Supplementary_file_1.docx]

**Supplementary Material**

Association Between the Difference in Cystatin C and Creatinine-Based eGFR and Risks of Multiple Cardiovascular Diseases: A Prospective Cohort Study

Zhiyu Qiao, Xinyi Liu, Hao Liu, Suwei Chen, Chengnan Li, Yipeng Ge, Haiou Hu, Junming Zhu

**Supplementary method**

**Supplementary Table**

**Table S1.** Lifestyle assessment

**Table S2.** Components of an ideal diet

**Table S3.** Baseline Characteristics Categorized by Race-Related eGFR_diff_

**Table S4.** Pearson's Correlation of Non-Race-Based eGFR_diff_ with Baseline Clinical Parameters

**Table S5.** Pearson's Correlation of Race-Related eGFR_diff_ with Baseline Clinical Parameters

**Table S6.** Incidence of Cardiovascular Disease and Its Components Across Three Categories of Race-Related eGFR_diff_ Levels

**Table S7.** Cardiovascular Disease Risk and Mortality Stratified by Race-Related eGFR_diff_ Levels

**Table S8.** Risk of Cardiovascular Disease Subtypes Stratified by Race-Related eGFR_diff_ Levels

**Table S9.** Association Between Non-Race-Based eGFR_diff_ and Cardiovascular Disease Risk and Mortality Adjusted for Baseline eGFR_cys_ or eGFR_cr-cys_

**Table S10.** Association Between Race-Related eGFR_diff_ and Cardiovascular Disease Risk and Mortality Adjusted for Baseline eGFR_cys_ or eGFR_cr-cys_

**Table S11.** Association Between Non-Race-Based eGFR_diff_ and Cardiovascular Disease Risk and Mortality Based on eGFR_cr_, eGFR_cys_, and eGFR_cr-cys_ Groups

**Table S12.** Association Between Race-Related eGFR_diff_ and Cardiovascular Disease Risk and Mortality Based on eGFR_cr_, eGFR_cys_, and eGFR_cr-cys_ Groups

**Table S13.** Sensitivity Analysis of Non-Race-Based eGFR_diff_ and Cardiovascular Disease Incidence and Mortality

**Table S14.** Sensitivity Analysis of Race-Related eGFR_diff_ and Cardiovascular Disease Incidence and Mortality

**Table S15.** Subgroup Analysis of The Association Between Cardiovascular Disease Incidence and eGFR_diff_

**Table S16.** Subgroup Analysis of The Association Between Cardiovascular Disease Mortality and eGFR_diff_

**Table S17.** Subgroup Analysis of The Association Between All-cause Mortality and eGFR_diff_

**Supplementary Figure**

**Figure S1.** Flowchart of study participants

**Figure S2.** Restrictive Cubic Spine Plot of Cardiovascular-related Morbidity According to Non-Race-Based eGFR_diff_

**Figure S3.** Restrictive Cubic Spine Plot of Cardiovascular-related Morbidity and Mortality According to Race-Related eGFR_diff_

**Table S1.** Lifestyle assessment

| **Lifestyle factor** | **Field IDs** | **Lifestyle assessment** |
| --- | --- | --- |
| Smoking | 1239 1249 2644 | Past or current smoking  Smoked at least 100 cigarettes in their lifetime |
| Drinking | 1558 1568 1578 1588 1598 1608 5364 | More than two drinks per day for men More than one drink per day for women |
| Sleep pattern | 1160  1180 1200 1210 1220 | For each component of the sleep pattern, a score of 1 is assigned to high-risk components, while low-risk components are assigned a score of 0. The total sleep pattern score ranges from 0 to 5, with scores of 0–1 indicating a low-risk category, 2–3 representing a medium-risk category, and 4–5 corresponding to high-risk sleep patterns. |
| Physical activity | 971 981 991 1001 2624 2634 3637 3647 | The cohort was categorized into light, moderate, and heavy physical activity levels based on the tertile distribution of weekly physical activity Metabolic Equivalent of Task (MET) values. |
| Sedentary time | 1070  1080 | The cohort was divided into mild, moderate, and severe levels of sedentary behavior based on the tertile distribution of sedentary time. |
| Diet | Supplementary table 2 | A diet consisting of five or more key components is considered a healthy diet. |

**Table S2.** Components of an ideal diet

| **Diet component** | **Field IDs** | **Intake goal** |
| --- | --- | --- |
| Fruit | 1309 1319 | ≥3 servings/day |
| Vegetable | 1289 1299 | ≥3 servings/day |
| Whole grains | 1438/1448 1458/1468 | ≥3 servings/day |
| Fish (Shell) | 1329 1339 | ≥2 servings/day |
| Dairy | 1408 1418 | ≥2 servings/day |
| Vegetable oils | 1428 1438 2654 | ≥2 servings/day |
| Refined grains | 1438/1448 1458/1468 | ≤2 servings/day |
| processed meats | 1349 3680 | ≤1 servings/day |
| Unprocessed meats | 1359 1369 1379 1389 3680 | ≤2 servings/day |
| Sugar-sweetened beverages | 6144 | No consumption |

**Table S3.** Baseline Characteristics Categorized by Race-Related eGFR_diff_

| **Characteristics** | **eGFR_diff_ Categories (mL/min/1.73 m^2^)** | | | **P-value** |
| --- | --- | --- | --- | --- |
|  | **Negative <-15 (N=41672)** | **Midrange -15 to 15 (N=228252)** | **Positive ≥15 (N=27216)** |  |
| **Demographics** | | | | |
| Age (years) | 57.6±7.8 | 55.9±8.1 | 53.3±7.9 | <0.001 |
| Male (%) | 19752 (47.7%) | 103923 (45.5%) | 12279 (45.1%) | <0.001 |
| White ethnicity or race (%) | 39082 (93.8%) | 219958 (96.4%) | 25079 (92.1%) | <0.001 |
| Townsend deprivation index | -1.2±3.1 | -1.7±2.9 | -1.6±2.9 | <0.001 |
| University or college degree (%) | 12178 (29.2%) | 84442 (37.0%) | 11015 (40.5%) | <0.001 |
| Employed, student, or retired (%) | 37413 (89.8%) | 210617 (92.3%) | 25133 (92.3%) | <0.001 |
| BMI | 29.0±5.3 | 26.7±4.1 | 26.0±3.6 | <0.001 |
| Grip strength (kg) | 39.5±10.9 | 31.5±10.9 | 33.6±11.1 | <0.001 |
| **Lifestyle** | | | | |
| Physical activity (%) | |  |  | <0.001 |
| Low | 17154 (41.2%) | 70086 (30.7%) | 6921 (25.4%) | |
| Moderate | 13477 (32.3%) | 77775 (34.1%) | 8980 (33.0%) | |
| High | 11041 (26.5%) | 80391 (35.2%) | 11315 (41.6%) | |
| Sleep patterns (%) | |  |  | <0.001 |
| Poor | 12397 (29.7%) | 80569 (35.3%) | 10183 (37.4%) | |
| Moderate | 26458 (63.5%) | 137202 (60.1%) | 15907 (58.4%) | |
| Good | 2817 (6.8%) | 10481 (4.6%) | 1126 (4.1%) | |
| No heavy alcohol (%) | 24040 (57.7%) | 105049 (46.0%) | 11490 (42.2%) | <0.001 |
| Never smoking (%) | 20011 (48.0%) | 131018 (57.4%) | 17028 (62.6%) | <0.001 |
| Healthy diet (%) | 4200 (10.1%) | 21512 (9.4%) | 2137 (7.9%) | <0.001 |
| Sedentary time (%) | |  |  | <0.001 |
| High | 9478 (22.7%) | 42602 (18.7%) | 4870 (17.9%) | |
| Moderate | 14468 (34.7%) | 75203 (32.9%) | 8586 (31.5%) | |
| Low | 17726 (42.5%) | 110447 (48.4%) | 13760 (50.6%) | |
| Healthy lifestyle score | 3.9±1.5 | 4.2±1.4 | 4.3±1.4 | <0.001 |
| **Medical history** | | | | |
| Chronic respiratory diseases (%) | 5861 (14.1%) | 27992 (12.3%) | 3242 (11.9%) | <0.001 |
| Chronic liver disease (%) | 291 (0.7%) | 499 (0.2%) | 30 (0.1%) | <0.001 |
| Hypertension (%) | 14535 (34.9%) | 57324 (25.1%) | 5442 (20.0%) | <0.001 |
| Hyperglycemia (%) | 4599 (11.0%) | 12593 (5.5%) | 1066 (3.9%) | <0.001 |
| Dyslipidemia (%) | 22207 (53.3%) | 84889 (37.2%) | 8018 (29.5%) | <0.001 |
| **Inflammation** | | | | |
| White blood cell count (x10^9/L) | 7.2±1.7 | 6.6±1.6 | 6.5±1.6 | <0.001 |
| Platelet count (x10^9/L) | 256±60.1 | 252±55.5 | 246±53.8 | <0.001 |
| Lymphocyte count (x10^9/L) | 2.1±0.7 | 1.9±0.6 | 1.9±0.5 | <0.001 |
| Neutrophil count (x10^9/L) | 4.4±1.4 | 4.1±1.3 | 3.9±1.3 | <0.001 |
| C-reactive protein (mg/L) | 2.5±1.9 | 1.7±1.6 | 1.4±1.4 | <0.001 |
| NLR | 2.3±1.1 | 2.3±1.1 | 2.3±1.0 | <0.001 |
| INFLA score | 1.7±5.9 | -0.5±5.9 | -1.5±6.0 | <0.001 |
| **Biochemical detection** | | | | |
| Albumin (g/L) | 45.0±2.6 | 45.4±2.5 | 45.6±2.6 | <0.001 |
| High-density lipoprotein (mmol/L) | 1.4±0.4 | 1.5±0.4 | 1.6±0.4 | <0.001 |
| Low-density lipoprotein (mmol/L) | 3.7±0.9 | 3.6±0.8 | 3.5±0.8 | <0.001 |
| Triglycerides (mmol/L) | 2.0±1.1 | 1.7±0.9 | 1.5±0.9 | <0.001 |
| UACR (mg/g) (%) | |  |  | <0.001 |
| <30 | 38688 (92.8%) | 215013 (94.2%) | 26227 (96.4%) | |
| 30 to 300 | 2795 (6.7%) | 12696 (5.6%) | 956 (3.5%) | |
| >300 | 189 (0.5%) | 543 (0.2%) | 33 (0.1%) |  |
| eGFR_cys_ (mL/min/1.73 m^2^) | 73.6±11.0 | 91.8±14.0 | 102±10.8 | <0.001 |
| eGFR_cr_ (mL/min/1.73 m^2^) | 95.4±10.2 | 92.2±12.5 | 79.6±11.6 | <0.001 |
| eGFR_diff_ (mL/min/1.73 m^2^) | -21.8±6.0 | -0.39±7.6 | 22.3±6.9 | <0.001 |

P values were determined using the ANOVA test for continuous variables and the chi-square test for categorical variables. Abbreviations: eGFR_diff_, the difference between cystatin C–based estimated glomerular filtration rate and creatinine-based estimated glomerular filtration rate; BMI, Body mass index; NLR, Neutrophil-to-Lymphocyte Ratio; INFLA score, Low-grade chronic inflammation score; UACR, Urinary albumin-creatinine ratio.

**Table S4.** Pearson's Correlation of Non-Race-Based eGFR_diff_ with Baseline Clinical Parameters

| **Variables** | **Pearson's correlation** | |
| --- | --- | --- |
|  | **Correlation coefficient** | **P-value** |
| Age (years) | -0.19 | <0.001 |
| Sex | -0.03 | <0.001 |
| Race | -0.04 | <0.001 |
| Education | 0.08 | <0.001 |
| Employed, student, or retired | 0.02 | <0.001 |
| Townsend deprivation index | -0.06 | <0.001 |
| BMI | -0.22 | <0.001 |
| Grip strength (kg) | 0.09 | <0.001 |
| ASM/BMI (m^2^) | 0.08 | <0.001 |
| **Lifestyle** |  |  |
| Physical activity | 0.11 | <0.001 |
| Sleep patterns | -0.06 | <0.001 |
| No heavy alcohol | -0.10 | <0.001 |
| Never smoking | 0.08 | <0.001 |
| Healthy diet | -0.02 | <0.001 |
| Sedentary time | 0.06 | <0.001 |
| Healthy lifestyle score | 0.06 | <0.001 |
| **Medical history** |  |  |
| Chronic respiratory diseases | -0.02 | <0.001 |
| Chronic liver disease | -0.03 | <0.001 |
| Hypertension | -0.11 | <0.001 |
| Hyperglycemia | -0.09 | <0.001 |
| Dyslipidemia | -0.16 | <0.001 |
| **Inflammation** |  |  |
| White blood cell count (x10^9/L) | -0.13 | <0.001 |
| Platelet count (x10^9/L) | -0.10 | <0.001 |
| Lymphocyte count (x10^9/L) | -0.10 | <0.001 |
| Neutrophil count (x10^9/L) | -0.10 | <0.001 |
| C-reactive protein (mg/L) | -0.22 | <0.001 |
| INFLA score | -0.17 | <0.001 |
| **Biochemical detection** |  |  |
| Albumin (g/L) | 0.08 | <0.001 |
| High-density lipoprotein (mmol/L) | 0.17 | <0.001 |
| Low-density lipoprotein (mmol/L) | -0.07 | <0.001 |
| Triglycerides (mmol/L) | -0.18 | <0.001 |
| UACR (mg/g) | -0.02 | <0.001 |

Abbreviations: eGFR_diff_, the difference between cystatin C–based estimated glomerular filtration rate and creatinine-based estimated glomerular filtration rate; BMI, Body mass index; NLR, Neutrophil-to-Lymphocyte Ratio; INFLA score, Low-grade chronic inflammation score.

**Table S5.** Pearson's Correlation of Race-Related eGFR_diff_ with Baseline Clinical Parameters

| **Variables** | **Pearson's correlation** | |
| --- | --- | --- |
|  | **Correlation coefficient** | **P-value** |
| Age (years) | -0.16 | <0.001 |
| Sex | -0.01 | <0.001 |
| Race | -0.01 | <0.001 |
| Education | 0.07 | <0.001 |
| Employed, student, or retired | 0.03 | <0.001 |
| Townsend deprivation index | -0.07 | <0.001 |
| BMI | -0.22 | <0.001 |
| Grip strength (kg) | 0.11 | <0.001 |
| ASM/BMI (m^2^) | 0.10 | <0.001 |
| **Lifestyle** |  |  |
| Physical activity | 0.12 | <0.001 |
| Sleep patterns | -0.06 | <0.001 |
| No heavy alcohol | -0.10 | <0.001 |
| Never smoking | 0.09 | <0.001 |
| Healthy diet | -0.02 | <0.001 |
| Sedentary time | 0.06 | <0.001 |
| Healthy lifestyle score | 0.06 | <0.001 |
| **Medical history** |  |  |
| Chronic respiratory diseases | -0.02 | <0.001 |
| Chronic liver disease | -0.03 | <0.001 |
| Hypertension | -0.11 | <0.001 |
| Hyperglycemia | -0.09 | <0.001 |
| Dyslipidemia | -0.16 | <0.001 |
| **Inflammation** |  |  |
| White blood cell count (x10^9/L) | -0.14 | <0.001 |
| Platelet count (x10^9/L) | -0.05 | <0.001 |
| Lymphocyte count (x10^9/L) | -0.11 | <0.001 |
| Neutrophil count (x10^9/L) | -0.10 | <0.001 |
| C-reactive protein (mg/L) | -0.22 | <0.001 |
| INFLA score | -0.17 | <0.001 |
| **Biochemical detection** |  |  |
| Albumin (g/L) | 0.08 | <0.001 |
| High-density lipoprotein (mmol/L) | 0.17 | <0.001 |
| Low-density lipoprotein (mmol/L) | -0.07 | <0.001 |
| Triglycerides (mmol/L) | -0.17 | <0.001 |
| UACR (mg/g) | -0.03 | <0.001 |

Abbreviations: eGFR_diff_, the difference between cystatin C–based estimated glomerular filtration rate and creatinine-based estimated glomerular filtration rate; BMI, Body mass index; NLR, Neutrophil-to-Lymphocyte Ratio; INFLA score, Low-grade chronic inflammation score.

**Table S6.** Incidence of Cardiovascular Disease and Its Components Across Three Categories of Race-Related eGFR_diff_ Levels

| **Outcomes** | **Total (N=297140)** | **eGFR_diff_ Categories (mL/min/1.73 m^2^)** | | |
| --- | --- | --- | --- | --- |
|  |  | **Negative <-15 (N=41672)** | **Midrange -15 to 15 (N=228252)** | **Positive ≥15 (N=27216)** |
| Incident CVD | 43315 (14.6%) | 8852 (21.2%) | 31856 (13.9%) | 2607 (9.6%) |
| Incidence rate^#^ | 10.48 (10.38-10.57) | 15.98 (15.66-16.31) | 9.98 (9.87-10.09) | 6.69 (6.44-6.95) |
| CVD mortality | 4634 (1.6%) | 1234 (2.9%) | 3206 (1.4%) | 194 (0.7%) |
| Incidence rate | 1.03 (1.00-1.06) | 1.97 (1.86-2.08) | 0.93 (0.89-0.96) | 0.47 (0.41-0.54) |
| All cause mortality | 19289 (6.5%) | 4545 (10.9%) | 13738 (6.0%) | 1006 (3.7%) |
| Incidence rate | 4.39 (4.33-4.46) | 7.52 (7.30-7.74) | 4.07 (3.99-4.13) | 2.48 (2.33-2.64) |
| **Incidence of CVD Components6** | | | | |
| Stroke | 5762 (1.9%) | 1238 (2.9%) | 4203 (1.8%) | 321 (1.2%) |
| Incidence rate | 1.32 (1.29-1.36) | 2.07 (1.96-2.19) | 1.25 (1.21-1.29) | 0.79 (0.71-0.89) |
| HF | 6451 (2.2%) | 1773 (4.3%) | 4414 (1.9%) | 264 (0.9%) |
| Incidence rate | 1.48 (1.44-1.52) | 2.97 (2.83-3.11) | 1.31 (1.28-1.35) | 0.65 (0.58-0.74) |
| AF | 15990 (5.4%) | 3384 (8.1%) | 11722 (5.1%) | 884 (3.2%) |
| Incidence rate | 3.72 (3.66-3.78) | 5.77 (5.58-5.97) | 3.54 (3.48-3.60) | 2.21 (2.07-2.36) |
| VHD | 8711 (2.9%) | 1836 (4.4%) | 6387 (2.8%) | 488 (1.8%) |
| Incidence rate | 2.00 (1.96-2.05) | 3.08 (2.94-3.22) | 1.91 (1.86-1.96) | 1.21 (1.11-1.32) |
| CAD | 18601 (6.3%) | 3930 (9.4%) | 13549 (5.9%) | 1122 (4.1%) |
| Incidence rate | 4.35 (4.29-4.42) | 6.77 (6.56-6.99) | 4.11 (4.05-4.18) | 2.82 (2.66-2.99) |
| AA | 1799 (0.6%) | 435 (1.0%) | 1285 (0.6%) | 79 (0.3%) |
| Incidence rate | 0.41 (0.39-0.43) | 0.72 (0.66-0.79) | 0.38 (0.36-0.40) | 0.19 (0.16-0.24) |
| PAD | 1871 (0.6%) | 536 (1.3%) | 1261 (0.6%) | 74 (0.3%) |
| Incidence rate | 0.43 (0.41-0.45) | 0.89 (0.82-0.97) | 0.37 (0.35-0.39) | 0.18 (0.15-0.23) |
| DVT | 2269 (0.8%) | 446 (1.1%) | 1645 (0.7%) | 178 (0.7%) |
| Incidence rate | 0.52 (0.49-0.54) | 0.74 (0.67-0.81) | 0.49 (0.46-0.51) | 0.44 (0.38-0.51) |
| PE | 4453 (1.5%) | 927 (2.2%) | 3243 (1.4%) | 283 (1.0%) |
| Incidence rate | 1.02 (0.99-1.05) | 1.54 (1.45-1.65) | 0.96 (0.93-0.99) | 0.70 (0.62-0.79) |
| AE | 578 (0.2%) | 156 (0.4%) | 400 (0.2%) | 22 (0.1%) |
| Incidence rate | 0.13 (0.12-0.14) | 0.26 (0.22-0.30) | 0.12 (0.11-0.13) | 0.05 (0.04-0.08) |

#The incidence rates of the corresponding diseases per 1,000 person-years were calculated.

Abbreviations: eGFR_diff_, the difference between cystatin C–based estimated glomerular filtration rate and creatinine-based estimated glomerular filtration rate; CVD, Cardiovascular disease; HF, Heart failure Heart failure; AF, Atrial fibrillation; VHD, Valvular heart disease; CAD, Coronary atherosclerotic heart disease; AA, Aortic aneurysm; PAD, Peripheral artery disease; DVT, Deep vein thrombosis; PE, Pulmonary embolism; AE, Arterial embolism.

**Table S7.** Cardiovascular Disease Risk and Mortality Stratified by Race-Related eGFR_diff_ Levels

| **Race-Related**  **eGFR_diff_** | **Model 1** | | **Model 2** | | **Model 3** | |
| --- | --- | --- | --- | --- | --- | --- |
|  | **HR**  **(95% CI)** | **P-value** | **HR**  **(95% CI)** | **P-value** | **HR**  **(95% CI)** | **P-value** |
| **Incident CVD** | | | | | | |
| Negative <-15 | 1.27 (1.24-1.30) | <0.001 | 1.25 (1.22-1.28) | <0.001 | 1.24 (1.21-1.27) | <0.001 |
| Midrange -15 to 15 | 1 (Reference) | | 1 (Reference) | | 1 (Reference) | |
| Positive ≥15 | 0.84 (0.81-0.88) | <0.001 | 0.85 (0.82-0.89) | <0.001 | 0.83 (0.79-0.86) | <0.001 |
| Per 10mL/min/1.73m^2^  increase | 0.90 (0.89-0.91) | <0.001 | 0.90 (0.89-0.91) | <0.001 | 0.90 (0.89-0.91) | <0.001 |
| **CVD mortality** | | | | | | |
| Negative <-15 | 1.57 (1.46-1.68) | <0.001 | 1.53 (1.43-1.63) | <0.001 | 1.50 (1.40-1.61) | <0.001 |
| Midrange -15 to 15 | 1 (Reference) | | 1 (Reference) | | 1 (Reference) | |
| Positive ≥15 | 0.69 (0.60-0.81) | <0.001 | 0.71 (0.61-0.82) | <0.001 | 0.67 (0.58-0.78) | <0.001 |
| Per 10mL/min/1.73m^2^  increase | 0.81 (0.79-0.82) | <0.001 | 0.81 (0.79-0.83) | <0.001 | 0.81 (0.79-0.83) | <0.001 |
| **All cause mortality** | | | | | | |
| Negative <-15 | 1.51 (1.46-1.57) | <0.001 | 1.48 (1.43-1.53) | <0.001 | 1.45 (1.39-1.50) | <0.001 |
| Midrange -15 to 15 | 1 (Reference) | | 1 (Reference) | | 1 (Reference) | |
| Positive ≥15 | 0.79 (0.74-0.85) | <0.001 | 0.80 (0.75-0.85) | <0.001 | 0.78 (0.73-0.84) | <0.001 |
| Per 10mL/min/1.73m^2^  increase | 0.83 (0.82-0.84) | <0.001 | 0.84 (0.83-0.85) | <0.001 | 0.84 (0.83-0.85) | <0.001 |

Model 1: Adjusted for age, sex, racial background, educational level, occupational status, Townsend deprivation index, and body mass index. Model 2: Further adjusted for healthy lifestyle score and comorbidities (chronic respiratory disease, chronic liver disease, hypertension, diabetes, and dyslipidemia). Model 3: Additionally adjusted for laboratory measurements (INFLA score, serum albumin, HDL-C, LDL-C, triglycerides, UACR, and eGFR_cr_). Abbreviation: eGFR_diff_, the difference between cystatin C–based estimated glomerular filtration rate and creatinine-based estimated glomerular filtration rate; CVD, Cardiovascular disease; HR, hazard ratio; CI, confidence interval; INFLA score, Low-grade chronic inflammation score; HDL-C, high-density lipoprotein cholesterol; LDL-C, low-density lipoprotein cholesterol; UACR, Urinary albumin-creatinine ratio; eGFR_cr_, creatinine-based estimated glomerular filtration rate.

**Table S8.** Risk of Cardiovascular Disease Subtypes Stratified by Race-Related eGFR_diff_ Levels

| **Characteristics** | **Negative <-15** | | **Midrange -15 to 15** | **Positive ≥15** | | **Per 10mL/min/1.73m^2^ increase** | |
| --- | --- | --- | --- | --- | --- | --- | --- |
|  | **HR**  **(95% CI)** | **P-value** | **HR**  **(95% CI)** | **HR**  **(95% CI)** | **P-value** | **HR**  **(95% CI)** | **P-value** |
| Stroke | 1.32 (1.24-1.41) | <0.001 | 1 (Reference) | 0.79 (0.71-0.89) | <0.001 | 0.89 (0.87-0.91) | <0.001 |
| HF | 1.56 (1.47-1.65) | <0.001 | 1 (Reference) | 0.66 (0.58-0.75) | <0.001 | 0.80 (0.78-0.82) | <0.001 |
| AF | 1.27 (1.22-1.32) | <0.001 | 1 (Reference) | 0.82 (0.77-0.88) | <0.001 | 0.89 (0.88-0.90) | <0.001 |
| VHD | 1.25 (1.18-1.32) | <0.001 | 1 (Reference) | 0.82 (0.74-0.90) | <0.001 | 0.89 (0.87-0.90) | <0.001 |
| CAD | 1.21 (1.16-1.25) | <0.001 | 1 (Reference) | 0.86 (0.81-0.92) | <0.001 | 0.91 (0.90-0.92) | <0.001 |
| AA | 1.47 (1.31-1.65) | <0.001 | 1 (Reference) | 0.70 (0.55-0.88) | 0.002 | 0.84 (0.81-0.88) | <0.001 |
| PAD | 1.60 (1.43-1.78) | <0.001 | 1 (Reference) | 0.68 (0.54-0.87) | 0.002 | 0.80 (0.77-0.84) | <0.001 |
| DVT | 1.26 (1.12-1.40) | <0.001 | 1 (Reference) | 0.93 (0.79-1.10) | 0.417 | 0.89 (0.86-0.93) | <0.001 |
| PE | 1.26 (1.17-1.37) | <0.001 | 1 (Reference) | 0.79 (0.70-0.90) | <0.001 | 0.89 (0.87-0.91) | <0.001 |
| AE | 1.59 (1.30-1.94) | <0.001 | 1 (Reference) | 0.58 (0.37-0.91) | 0.017 | 0.82 (0.77-0.89) | <0.001 |

This analysis adjusted for age, sex, racial background, educational level, occupational status, Townsend deprivation index, body mass index, healthy lifestyle score, comorbidities (chronic respiratory disease, chronic liver disease, hypertension, diabetes, and dyslipidemia), and laboratory measurements (INFLA score, serum albumin, HDL-C, LDL-C, triglycerides, UACR, and eGFR_cr_). Abbreviation: eGFR_diff_, the difference between cystatin C–based estimated glomerular filtration rate and creatinine-based estimated glomerular filtration rate; HR, hazard ratio; CI, confidence interval; INFLA score, Low-grade chronic inflammation score; HDL-C, high-density lipoprotein cholesterol; LDL-C, low-density lipoprotein cholesterol; UACR, Urinary albumin-creatinine ratio; eGFR_cr_, creatinine-based estimated glomerular filtration rate; HF, Heart failure Heart failure; AF, Atrial fibrillation; VHD, Valvular heart disease; CAD, Coronary atherosclerotic heart disease; AA, Aortic aneurysm; PAD, Peripheral artery disease; DVT, Deep vein thrombosis; PE, Pulmonary embolism; AE, Arterial embolism.

**Table S9.** Association Between Non-Race-Based eGFR_diff_ and Cardiovascular Disease Risk and Mortality Adjusted for Baseline eGFR_cys_ or eGFR_cr-cys_

| **Characteristics** | **HR (95% CI) (Model + eGFR_cys_)** | | | | **HR (95% CI) (Model + eGFR_cr-cys_)** | | | |
| --- | --- | --- | --- | --- | --- | --- | --- | --- |
|  | **Negative <-15** | **Midrange -15 to 15** | **Positive ≥15** | **Per 10mL/min/1.73m^2^ increase** | **Negative <-15** | **Midrange -15 to 15** | **Positive ≥15** | **Per 10mL/min/1.73m^2^ increase** |
| Incident CVD | 1.14 (1.11-1.17) | 1 (Reference) | 0.94 (0.89-0.98) | 0.94 (0.93-0.94) | 1.15 (1.13-1.18) | 1 (Reference) | 0.90 (0.85-0.94) | 0.93 (0.92-0.94) |
| CVD mortality | 1.30 (1.21-1.39) | 1 (Reference) | 0.79 (0.65-0.97) | 0.87 (0.84-0.89) | 1.31 (1.23-1.40) | 1 (Reference) | 0.73 (0.60-0.89) | 0.87 (0.84-0.89) |
| All cause mortality | 1.33 (1.29-1.38) | 1 (Reference) | 0.83 (0.76-0.90) | 0.87 (0.85-0.88) | 1.33 (1.28-1.37) | 1 (Reference) | 0.80 (0.73-0.87) | 0.87 (0.86-0.88) |
| **Subtypes of CVD** | | | | | | | | |
| Stroke | 1.17 (1.10-1.25) | 1 (Reference) | 0.88 (0.76-1.02) | 0.92 (0.90-0.94) | 1.18 (1.11-1.25) | 1 (Reference) | 0.85 (0.73-0.98) | 0.92 (0.90-0.94) |
| HF | 1.39 (1.31-1.47) | 1 (Reference) | 0.82 (0.69-0.96) | 0.85 (0.83-0.87) | 1.41 (1.33-1.48) | 1 (Reference) | 0.76 (0.65-0.89) | 0.85 (0.83-0.86) |
| AF | 1.20 (1.15-1.24) | 1 (Reference) | 0.91 (0.83-0.99) | 0.92 (0.90-0.93) | 1.21 (1.17-1.25) | 1 (Reference) | 0.88 (0.81-0.97) | 0.91 (0.90-0.93) |
| VHD | 1.15 (1.09-1.21) | 1 (Reference) | 0.91 (0.80-1.03) | 0.93 (0.91-0.95) | 1.16 (1.11-1.22) | 1 (Reference) | 0.86 (0.76-0.98) | 0.92 (0.91-0.94) |
| CAD | 1.11 (1.07-1.15) | 1 (Reference) | 0.95 (0.88-1.03) | 0.95 (0.94-0.96) | 1.13 (1.09-1.16) | 1 (Reference) | 0.91 (0.84-0.98) | 0.94 (0.93-0.96) |
| AA | 1.18 (1.06-1.32) | 1 (Reference) | 0.94 (0.71-1.26) | 0.89 (0.85-0.93) | 1.24 (1.12-1.37) | 1 (Reference) | 0.88 (0.66-1.16) | 0.88 (0.84-0.91) |
| PAD | 1.50 (1.34-1.67) | 1 (Reference) | 0.72 (0.52-0.98) | 0.83 (0.79-0.87) | 1.47 (1.33-1.62) | 1 (Reference) | 0.70 (0.51-0.96) | 0.84 (0.80-0.87) |
| DVT | 1.15 (1.04-1.28) | 1 (Reference) | 1.32 (1.09-1.61) | 0.99 (0.96-1.03) | 1.15 (1.05-1.27) | 1 (Reference) | 1.21 (0.99-1.47) | 0.98 (0.95-1.01) |
| PE | 1.08 (1.01-1.16) | 1 (Reference) | 1.09 (0.93-1.27) | 0.98 (0.95-1.01) | 1.11 (1.04-1.19) | 1 (Reference) | 0.98 (0.84-1.14) | 0.96 (0.94-0.98) |
| AE | 1.31 (1.07-1.60) | 1 (Reference) | 0.44 (0.23-0.86) | 0.84 (0.77-0.90) | 1.27 (1.06-1.53) | 1 (Reference) | 0.43 (0.22-0.83) | 0.85 (0.80-0.92) |

This analysis adjusted for age, sex, racial background, educational level, occupational status, Townsend deprivation index, body mass index, healthy lifestyle score, comorbidities (chronic respiratory disease, chronic liver disease, hypertension, diabetes, and dyslipidemia), and laboratory measurements (eGFR_cys_ or non-race-based eGFR_cr-cys_, INFLA score, serum albumin, HDL-C, LDL-C, triglycerides, and UACR). Abbreviation: eGFR_diff_, the difference between cystatin C–based estimated glomerular filtration rate and creatinine-based estimated glomerular filtration rate; HR, hazard ratio; CI, confidence interval; INFLA score, Low-grade chronic inflammation score; HDL-C, high-density lipoprotein cholesterol; LDL-C, low-density lipoprotein cholesterol; UACR, Urinary albumin-creatinine ratio.

**Table S10.** Association Between Race-Related eGFR_diff_ and Cardiovascular Disease Risk and Mortality Adjusted for Baseline eGFR_cys_ or eGFR_cr-cys_

| **Characteristics** | **HR (95% CI) (Model + eGFR_cys_)** | | | | **HR (95% CI) (Model + eGFR_cr-cys_)** | | | |
| --- | --- | --- | --- | --- | --- | --- | --- | --- |
|  | **Negative <-15** | **Midrange -15 to 15** | **Positive ≥15** | **Per 10mL/min/1.73m^2^ increase** | **Negative <-15** | **Midrange -15 to 15** | **Positive ≥15** | **Per 10mL/min/1.73m^2^ increase** |
| Incident CVD | 1.15 (1.12-1.18) | 1 (Reference) | 0.90 (0.86-0.94) | 0.93 (0.92-0.94) | 1.17 (1.14-1.20) | 1 (Reference) | 0.86 (0.83-0.90) | 0.93 (0.92-0.94) |
| CVD mortality | 1.32 (1.23-1.42) | 1 (Reference) | 0.79 (0.68-0.91) | 0.86 (0.84-0.88) | 1.37 (1.28-1.47) | 1 (Reference) | 0.73 (0.63-0.84) | 0.85 (0.83-0.87) |
| All cause mortality | 1.35 (1.30-1.40) | 1 (Reference) | 0.84 (0.79-0.89) | 0.86 (0.85-0.87) | 1.38 (1.33-1.43) | 1 (Reference) | 0.80 (0.75-0.86) | 0.86 (0.85-0.87) |
| **Subtypes of CVD** | | | | | | | | |
| Stroke | 1.23 (1.15-1.32) | 1 (Reference) | 0.85 (0.76-0.96) | 0.92 (0.89-0.94) | 1.26 (1.18-1.35) | 1 (Reference) | 0.83 (0.74-0.93) | 0.91 (0.89-0.93) |
| HF | 1.39 (1.31-1.48) | 1 (Reference) | 0.76 (0.67-0.86) | 0.84 (0.82-0.86) | 1.44 (1.36-1.53) | 1 (Reference) | 0.70 (0.62-0.80) | 0.84 (0.82-0.85) |
| AF | 1.20 (1.15-1.25) | 1 (Reference) | 0.87 (0.81-0.94) | 0.91 (0.90-0.93) | 1.22 (1.18-1.27) | 1 (Reference) | 0.84 (0.79-0.90) | 0.91 (0.90-0.92) |
| VHD | 1.16 (1.09-1.23) | 1 (Reference) | 0.89 (0.81-0.98) | 0.92 (0.90-0.94) | 1.19 (1.12-1.25) | 1 (Reference) | 0.85 (0.78-0.93) | 0.92 (0.90-0.93) |
| CAD | 1.12 (1.08-1.17) | 1 (Reference) | 0.94 (0.88-0.99) | 0.95 (0.93-0.96) | 1.15 (1.11-1.19) | 1 (Reference) | 0.90 (0.84-0.95) | 0.94 (0.93-0.95) |
| AA | 1.31 (1.17-1.50) | 1 (Reference) | 0.78 (0.62-0.99) | 0.89 (0.85-0.93) | 1.38 (1.23-1.54) | 1 (Reference) | 0.73 (0.58-0.92) | 0.87 (0.84-0.91) |
| PAD | 1.50 (1.34-1.68) | 1 (Reference) | 0.72 (0.56-0.91) | 0.82 (0.79-0.86) | 1.53 (1.38-1.71) | 1 (Reference) | 0.69 (0.55-0.88) | 0.82 (0.79-0.86) |
| DVT | 1.05 (0.93-1.17) | 1 (Reference) | 1.18 (1.01-1.38) | 0.99 (0.95-1.03) | 1.08 (0.97-1.21) | 1 (Reference) | 1.07 (0.91-1.25) | 0.97 (0.94-1.00) |
| PE | 1.07 (0.98-1.16) | 1 (Reference) | 0.99 (0.88-1.13) | 0.98 (0.96-1.01) | 1.11 (1.03-1.20) | 1 (Reference) | 0.90 (0.80-1.02) | 0.95 (0.93-0.98) |
| AE | 1.53 (1.24-1.89) | 1 (Reference) | 0.61 (0.40-0.95) | 0.83 (0.77-0.90) | 1.51 (1.24-1.84) | 1 (Reference) | 0.60 (0.39-0.93) | 0.85 (0.79-0.91) |

This analysis adjusted for age, sex, racial background, educational level, occupational status, Townsend deprivation index, body mass index, healthy lifestyle score, comorbidities (chronic respiratory disease, chronic liver disease, hypertension, diabetes, and dyslipidemia), and laboratory measurements (eGFR_cys_ or race-related eGFR_cr-cys_, INFLA score, serum albumin, HDL-C, LDL-C, triglycerides, and UACR). Abbreviation: eGFR_diff_, the difference between cystatin C–based estimated glomerular filtration rate and creatinine-based estimated glomerular filtration rate; HR, hazard ratio; CI, confidence interval; INFLA score, Low-grade chronic inflammation score; HDL-C, high-density lipoprotein cholesterol; LDL-C, low-density lipoprotein cholesterol; UACR, Urinary albumin-creatinine ratio.

**Table S11.** Association Between Non-Race-Based eGFR_diff_ and Cardiovascular Disease Risk and Mortality Based on eGFR_cr_, eGFR_cys_, and eGFR_cr-cys_ Groups

|  | **eGFR_diff_ (Incident CVD)** | | | **eGFR_diff_ (CVD mortality)** | | | **eGFR_diff_ (All cause mortality)** | | |
| --- | --- | --- | --- | --- | --- | --- | --- | --- | --- |
|  | **N** | **HR (95% CI)** | **P-value** | **N** | **HR (95% CI)** | **P-value** | **N** | **HR (95% CI)** | **P-value** |
| **Classification by Non-Race-Based eGFR_cr_** | | | | | | | | | |
| ≥90 mL/min/1.73 m^2^ | 27727 | 0.90 (0.89-0.91) | <0.001 | 2803 | 0.82 (0.79-0.85) | <0.001 | 12324 | 0.84 (0.82-0.85) | <0.001 |
| 60-89 mL/min/1.73 m^2^ | 14632 | 0.89 (0.88-0.90) | <0.001 | 1648 | 0.78 (0.75-0.82) | <0.001 | 6434 | 0.84 (0.82-0.85) | <0.001 |
| 45-59 mL/min/1.73 m^2^ | 735 | 0.88 (0.83-0.93) | <0.001 | 124 | 0.81 (0.69-0.95) | 0.008 | 396 | 0.81 (0.75-0.88) | <0.001 |
| <45 mL/min/1.73 m^2^ | 221 | 0.70 (0.59-0.84) | <0.001 | 59 | 0.45 (0.28-0.73) | 0.001 | 135 | 0.57 (0.43-0.74) | <0.001 |
| **Classification by eGFR_cys_** | | | | | | | | | |
| ≥90 mL/min/1.73 m^2^ | 15325 | 0.94 (0.92-0.97) | <0.001 | 1256 | 0.91 (0.84-1.00) | 0.059 | 6325 | 0.91 (0.87-0.95) | <0.001 |
| 60-89 mL/min/1.73 m^2^ | 25022 | 0.89 (0.87-0.90) | <0.001 | 2805 | 0.82 (0.77-0.86) | <0.001 | 11257 | 0.82 (0.80-0.84) | <0.001 |
| 45-59 mL/min/1.73 m^2^ | 2439 | 0.78 (0.70-0.87) | <0.001 | 428 | 0.56 (0.44-0.72) | <0.001 | 1358 | 0.64 (0.56-0.73) | <0.001 |
| <45 mL/min/1.73 m^2^ | 529 | 0.85 (0.74-0.98) | 0.026 | 145 | 0.76 (0.59-0.98) | 0.032 | 349 | 0.73 (0.62-0.86) | <0.001 |
| **Classification by Non-Race-Based eGFR_cr-cys_** | | | | | | | | | |
| ≥90 mL/min/1.73 m^2^ | 23973 | 0.93 (0.92-0.95) | <0.001 | 2179 | 0.88 (0.84-0.92) | <0.001 | 10160 | 0.90 (0.88-0.92) | <0.001 |
| 60-89 mL/min/1.73 m^2^ | 18251 | 0.87 (0.86-0.89) | <0.001 | 2215 | 0.77 (0.73-0.81) | <0.001 | 8478 | 0.81 (0.79-0.83) | <0.001 |
| 45-59 mL/min/1.73 m^2^ | 815 | 0.85 (0.75-0.97) | 0.014 | 167 | 0.68 (0.51-0.91) | 0.011 | 472 | 0.70 (0.59-0.84) | <0.001 |
| <45 mL/min/1.73 m^2^ | 276 | 0.84 (0.70-1.00) | 0.059 | 73 | 0.76 (0.54-1.07) | 0.118 | 179 | 0.72 (0.58-0.89) | 0.002 |

This analysis adjusted for age, sex, racial background, educational level, occupational status, Townsend deprivation index, body mass index, healthy lifestyle score, comorbidities (chronic respiratory disease, chronic liver disease, hypertension, diabetes, and dyslipidemia), and laboratory measurements (non-race-based eGFR_cr_, INFLA score, serum albumin, HDL-C, LDL-C, triglycerides, and UACR). Abbreviation: eGFR_diff_, the difference between cystatin C–based estimated glomerular filtration rate and creatinine-based estimated glomerular filtration rate; HR, hazard ratio; CI, confidence interval; INFLA score, Low-grade chronic inflammation score; HDL-C, high-density lipoprotein cholesterol; LDL-C, low-density lipoprotein cholesterol; UACR, Urinary albumin-creatinine ratio.

**Table S12.** Association Between Race-Related eGFR_diff_ and Cardiovascular Disease Risk and Mortality Based on eGFR_cr_, eGFR_cys_, and eGFR_cr-cys_ Groups

|  | **eGFR_diff_ (Incident CVD)** | | | **eGFR_diff_ (CVD mortality)** | | | **eGFR_diff_ (All cause mortality)** | | |
| --- | --- | --- | --- | --- | --- | --- | --- | --- | --- |
|  | **N** | **HR (95% CI)** | **P-value** | **N** | **HR (95% CI)** | **P-value** | **N** | **HR (95% CI)** | **P-value** |
| **Classification by Race-Related eGFR_cr_** | | | | | | | | | |
| ≥90 mL/min/1.73 m^2^ | 22294 | 0.90 (0.89-0.91) | <0.001 | 2213 | 0.83 (0.80-0.86) | <0.001 | 9927 | 0.84 (0.82-0.85) | <0.001 |
| 60-89 mL/min/1.73 m^2^ | 19602 | 0.90 (0.89-0.90) | <0.001 | 2165 | 0.79 (0.76-0.82) | <0.001 | 8594 | 0.83 (0.82-0.85) | <0.001 |
| 45-59 mL/min/1.73 m^2^ | 1141 | 0.87 (0.83-0.91) | <0.001 | 185 | 0.78 (0.69-0.89) | <0.001 | 604 | 0.83 (0.77-0.88) | <0.001 |
| <45 mL/min/1.73 m^2^ | 278 | 0.75 (0.65-0.87) | <0.001 | 71 | 0.53 (0.37-0.77) | <0.001 | 164 | 0.61 (0.49-0.76) | <0.001 |
| **Classification by eGFR_cys_** | | | | | | | | | |
| ≥90 mL/min/1.73 m^2^ | 15325 | 0.94 (0.92-0.96) | <0.001 | 1256 | 0.91 (0.83-0.99) | 0.039 | 6325 | 0.90 (0.87-0.94) | <0.001 |
| 60-89 mL/min/1.73 m^2^ | 25022 | 0.89 (0.87-0.90) | <0.001 | 2805 | 0.81 (0.77-0.86) | <0.001 | 11257 | 0.82 (0.80-0.84) | <0.001 |
| 45-59 mL/min/1.73 m^2^ | 2439 | 0.78 (0.70-0.87) | <0.001 | 428 | 0.56 (0.44-0.72) | <0.001 | 1358 | 0.64 (0.56-0.73) | <0.001 |
| <45 mL/min/1.73 m^2^ | 529 | 0.85 (0.74-0.98) | 0.027 | 145 | 0.76 (0.59-0.98) | 0.035 | 349 | 0.73 (0.62-0.87) | <0.001 |
| **Classification by Race-Related eGFR_cr-cys_** | | | | | | | | | |
| ≥90 mL/min/1.73 m^2^ | 17508 | 0.94 (0.92-0.95) | <0.001 | 1540 | 0.87 (0.82-0.92) | <0.001 | 7341 | 0.89 (0.87-0.92) | <0.001 |
| 60-89 mL/min/1.73 m^2^ | 24234 | 0.88 (0.87-0.89) | <0.001 | 2767 | 0.79 (0.76-0.83) | <0.001 | 11044 | 0.82 (0.80-0.84) | <0.001 |
| 45-59 mL/min/1.73 m^2^ | 1226 | 0.80 (0.73-0.88) | <0.001 | 240 | 0.63 (0.51-0.79) | <0.001 | 682 | 0.67 (0.59-0.77) | <0.001 |
| <45 mL/min/1.73 m^2^ | 347 | 0.84 (0.73-0.97) | 0.019 | 87 | 0.78 (0.57-1.05) | 0.104 | 222 | 0.73 (0.61-0.88) | <0.001 |

This analysis adjusted for age, sex, racial background, educational level, occupational status, Townsend deprivation index, body mass index, healthy lifestyle score, comorbidities (chronic respiratory disease, chronic liver disease, hypertension, diabetes, and dyslipidemia), and laboratory measurements (race-related eGFR_cr_, INFLA score, serum albumin, HDL-C, LDL-C, triglycerides, and UACR). Abbreviation: eGFR_diff_, the difference between cystatin C–based estimated glomerular filtration rate and creatinine-based estimated glomerular filtration rate; HR, hazard ratio; CI, confidence interval; INFLA score, Low-grade chronic inflammation score; HDL-C, high-density lipoprotein cholesterol; LDL-C, low-density lipoprotein cholesterol; UACR, Urinary albumin-creatinine ratio.

**Table S13.** Sensitivity Analysis of Non-Race-Based eGFR_diff_ and Cardiovascular Disease Incidence and Mortality

| **Analyses** | **eGFR_diff_ (Incident CVD)** | | **eGFR_diff_ (CVD mortality)** | | **eGFR_diff_ (All cause mortality)** | |
| --- | --- | --- | --- | --- | --- | --- |
|  | **HR (95% CI)** | **P-value** | **HR (95% CI)** | **P-value** | **HR (95% CI)** | **P-value** |
| **Classification using tertile group of non-race-based eGFR_diff_** | | | | | | |
| Tertile 1 (<-10) | 1.17 (1.14-1.20) | <0.001 | 1.32 (1.23-1.42) | <0.001 | 1.28 (1.24-1.32) | <0.001 |
| Tertile 2 (-10 to 0) | 1 (Reference) |  | 1 (Reference) |  | 1 (Reference) |  |
| Tertile 1 (>0) | 0.91 (0.89-0.94) | <0.001 | 0.80 (0.73-0.87) | <0.001 | 0.87 (0.84-0.91) | <0.001 |
| **Additional adjustment for sarcopenia** | | | | | | |
| Negative <-15 | 1.22 (1.20-1.25) | <0.001 | 1.47 (1.37-1.56) | <0.001 | 1.41 (1.36-1.45) | <0.001 |
| Midrange -15 to 15 | 1 (Reference) |  | 1 (Reference) |  | 1 (Reference) |  |
| Positive ≥15 | 0.86 (0.81-0.90) | <0.001 | 0.67 (0.55-0.81) | <0.001 | 0.77 (0.70-0.84) | <0.001 |
| Per 10mL/min/1.73m^2^ increase | 0.90 (0.89-0.91) | <0.001 | 0.81 (0.79-0.83) | <0.001 | 0.84 (0.83-0.85) | <0.001 |
| **Simultaneous adjustment for lifestyle components and chronic inflammation detection indicators** | | | | | | |
| Negative <-15 | 1.20 (1.17-1.22) | <0.001 | 1.42 (1.33-1.52) | <0.001 | 1.36 (1.32-1.41) | <0.001 |
| Midrange -15 to 15 | 1 (Reference) |  | 1 (Reference) |  | 1 (Reference) |  |
| Positive ≥15 | 0.86 (0.82-0.91) | <0.001 | 0.68 (0.55-0.82) | <0.001 | 0.78 (0.72-0.86) | <0.001 |
| Per 10mL/min/1.73m^2^ increase | 0.91 (0.90-0.92) | <0.001 | 0.82 (0.80-0.84) | <0.001 | 0.85 (0.84-0.86) | <0.001 |
| **Excludes individuals with comorbidities** | | | | | | |
| Negative <-15 | 1.24 (1.19-1.30) | <0.001 | 1.57 (1.36-1.81) | <0.001 | 1.40 (1.31-1.49) | <0.001 |
| Midrange -15 to 15 | 1 (Reference) |  | 1 (Reference) |  | 1 (Reference) |  |
| Positive ≥15 | 0.89 (0.81-0.98) | 0.013 | 0.73 (0.50-1.07) | 0.111 | 0.85 (0.73-0.98) | 0.025 |
| Per 10mL/min/1.73m^2^ increase | 0.90 (0.88-0.91) | <0.001 | 0.78 (0.74-0.83) | <0.001 | 0.86 (0.84-0.88) | <0.001 |
| **Excluding events that occurred within the first three years of follow-up** | | | | | | |
| Negative <-15 | 1.21 (1.18-1.24) | <0.001 | 1.46 (1.37-1.56) | <0.001 | 1.40 (1.35-1.45) | <0.001 |
| Midrange -15 to 15 | 1 (Reference) |  | 1 (Reference) |  | 1 (Reference) |  |
| Positive ≥15 | 0.87 (0.82-0.92) | <0.001 | 0.69 (0.56-0.85) | <0.001 | 0.77 (0.71-0.85) | <0.001 |
| Per 10mL/min/1.73m^2^ increase | 0.90 (0.89-0.91) | <0.001 | 0.81 (0.79-0.83) | <0.001 | 0.84 (0.83-0.86) | <0.001 |
| **Multiple imputation** | | | | | | |
| Negative <-15 | 1.22 (1.20-1.25) | <0.001 | 1.46 (1.37-1.56) | <0.001 | 1.41 (1.36-1.45) | <0.001 |
| Midrange -15 to 15 | 1 (Reference) |  | 1 (Reference) |  | 1 (Reference) |  |
| Positive ≥15 | 0.85 (0.81-0.90) | <0.001 | 0.66 (0.54-0.80) | <0.001 | 0.77 (0.70-0.84) | <0.001 |
| Per 10mL/min/1.73m^2^ increase | 0.90 (0.89-0.91) | <0.001 | 0.81 (0.79-0.83) | <0.001 | 0.84 (0.83-0.85) | <0.001 |

This analysis adjusted for age, sex, racial background, educational level, occupational status, Townsend deprivation index, body mass index, healthy lifestyle score, comorbidities (chronic respiratory disease, chronic liver disease, hypertension, diabetes, and dyslipidemia), and laboratory measurements (race-related eGFR_cr_, INFLA score, serum albumin, HDL-C, LDL-C, triglycerides, and UACR). Abbreviation: eGFR_diff_, the difference between cystatin C–based estimated glomerular filtration rate and creatinine-based estimated glomerular filtration rate; HR, hazard ratio; CI, confidence interval; INFLA score, Low-grade chronic inflammation score; HDL-C, high-density lipoprotein cholesterol; LDL-C, low-density lipoprotein cholesterol; UACR, Urinary albumin-creatinine ratio.

**Table S14.** Sensitivity Analysis of Race-Related eGFR_diff_ and Cardiovascular Disease Incidence and Mortality

| **Analyses** | **eGFR_diff_ (Incident CVD)** | | **eGFR_diff_ (CVD mortality)** | | **eGFR_diff_ (All cause mortality)** | |
| --- | --- | --- | --- | --- | --- | --- |
|  | **HR (95% CI)** | **P-value** | **HR (95% CI)** | **P-value** | **HR (95% CI)** | **P-value** |
| **Classification using tertile group of race-related eGFR_diff_** | | | | | | |
| Tertile 1 (<-6) | 1.17 (1.14-1.19) | <0.001 | 1.34 (1.25-1.43) | <0.001 | 1.30 (1.25-1.34) | <0.001 |
| Tertile 2 (-6 to 4) | 1 (Reference) |  | 1 (Reference) |  | 1 (Reference) |  |
| Tertile 1 (>4) | 0.90 (0.88-0.93) | <0.001 | 0.80 (0.73-0.87) | <0.001 | 0.87 (0.83-0.90) | <0.001 |
| **Additional adjustment for sarcopenia** | | | | | | |
| Negative <-15 | 1.24 (1.21-1.27) | <0.001 | 1.51 (1.40-1.61) | <0.001 | 1.45 (1.40-1.50) | <0.001 |
| Midrange -15 to 15 | 1 (Reference) |  | 1 (Reference) |  | 1 (Reference) |  |
| Positive ≥15 | 0.83 (0.79-0.86) | <0.001 | 0.67 (0.58-0.78) | <0.001 | 0.78 (0.73-0.84) | <0.001 |
| Per 10mL/min/1.73m^2^ increase | 0.90 (0.89-0.91) | <0.001 | 0.81 (0.79-0.83) | <0.001 | 0.84 (0.83-0.85) | <0.001 |
| **Simultaneous adjustment for lifestyle components and chronic inflammation detection indicators** | | | | | | |
| Negative <-15 | 1.21 (1.18-1.24) | <0.001 | 1.46 (1.36-1.56) | <0.001 | 1.40 (1.35-1.45) | <0.001 |
| Midrange -15 to 15 | 1 (Reference) |  | 1 (Reference) |  | 1 (Reference) |  |
| Positive ≥15 | 0.84 (0.80-0.87) | <0.001 | 0.68 (0.59-0.79) | <0.001 | 0.80 (0.75-0.86) | <0.001 |
| Per 10mL/min/1.73m^2^ increase | 0.91 (0.90-0.91) | <0.001 | 0.82 (0.79-0.84) | <0.001 | 0.85 (0.84-0.86) | <0.001 |
| **Excludes individuals with comorbidities** | | | | | | |
| Negative <-15 | 1.30 (1.23-1.37) | <0.001 | 1.74 (1.47-2.04) | <0.001 | 1.52 (1.41-1.63) | <0.001 |
| Midrange -15 to 15 | 1 (Reference) |  | 1 (Reference) |  | 1 (Reference) |  |
| Positive ≥15 | 0.88 (0.82-0.95) | <0.001 | 0.63 (0.47-0.86) | 0.003 | 0.88 (0.79-0.98) | 0.026 |
| Per 10mL/min/1.73m^2^ increase | 0.90 (0.88-0.91) | <0.001 | 0.78 (0.74-0.83) | <0.001 | 0.85 (0.83-0.87) | <0.001 |
| **Excluding events that occurred within the first three years of follow-up** | | | | | | |
| Negative <-15 | 1.23 (1.19-1.26) | <0.001 | 1.50 (1.39-1.61) | <0.001 | 1.43 (1.38-1.48) | <0.001 |
| Midrange -15 to 15 | 1 (Reference) |  | 1 (Reference) |  | 1 (Reference) |  |
| Positive ≥15 | 0.83 (0.80-0.87) | <0.001 | 0.69 (0.59-0.80) | <0.001 | 0.78 (0.73-0.84) | <0.001 |
| Per 10mL/min/1.73m^2^ increase | 0.90 (0.89-0.91) | <0.001 | 0.80 (0.78-0.83) | <0.001 | 0.84 (0.83-0.85) | <0.001 |
| **Multiple imputation** | | | | | | |
| Negative <-15 | 1.24 (1.21-1.27) | <0.001 | 1.52 (1.41-1.63) | <0.001 | 1.45 (1.40-1.50) | <0.001 |
| Midrange -15 to 15 | 1 (Reference) |  | 1 (Reference) |  | 1 (Reference) |  |
| Positive ≥15 | 0.83 (0.79-0.86) | <0.001 | 0.67 (0.57-0.77) | <0.001 | 0.78 (0.73-0.83) | <0.001 |
| Per 10mL/min/1.73m^2^ increase | 0.90 (0.89-0.90) | <0.001 | 0.80 (0.78-0.82) | <0.001 | 0.83 (0.82-0.85) | <0.001 |

This analysis adjusted for age, sex, racial background, educational level, occupational status, Townsend deprivation index, body mass index, healthy lifestyle score, comorbidities (chronic respiratory disease, chronic liver disease, hypertension, diabetes, and dyslipidemia), and laboratory measurements (race-related eGFR_cr_, INFLA score, serum albumin, HDL-C, LDL-C, triglycerides, and UACR). Abbreviation: eGFR_diff_, the difference between cystatin C–based estimated glomerular filtration rate and creatinine-based estimated glomerular filtration rate; HR, hazard ratio; CI, confidence interval; INFLA score, Low-grade chronic inflammation score; HDL-C, high-density lipoprotein cholesterol; LDL-C, low-density lipoprotein cholesterol; UACR, Urinary albumin-creatinine ratio.

**Table S15.** Subgroup Analysis of The Association Between Cardiovascular Disease Incidence and eGFR_diff_

| **Characteristics** | **Non-Race-Based eGFR_diff_ (Incident CVD)** | | | | **Race-Related eGFR_diff_ (Incident CVD)** | | | |
| --- | --- | --- | --- | --- | --- | --- | --- | --- |
|  | **Negative <-15** | **Midrange -15 to 15** | **Positive ≥ 15** | **Per 10mL/min/1.73m2 increase** | **Negative <-15** | **Midrange -15 to 15** | **Positive ≥ 15** | **Per 10mL/min/1.73m2 increase** |
| **Sex** | | | | | | | | |
| Male | 1.20 (1.17-1.24) | 1 (Reference) | 0.86 (0.81-0.93) | 0.91 (0.90-0.92) | 1.23 (1.19-1.27) | 1 (Reference) | 0.85 (0.80-0.89) | 0.91 (0.90-0.92) |
| Female | 1.26 (1.21-1.30) | 1 (Reference) | 0.83 (0.77-0.91) | 0.88 (0.87-0.90) | 1.26 (1.21-1.31) | 1 (Reference) | 0.79 (0.73-0.84) | 0.88 (0.87-0.89) |
| P for interaction | 0.548 | | | | 0.122 | | | |
| **Age** | | | | | | | | |
| ≥60 years | 1.28 (1.25-1.31) | 1 (Reference) | 0.73 (0.68-0.79) | 0.87 (0.87-0.88) | 1.29 (1.25-1.33) | 1 (Reference) | 0.73 (0.69-0.78) | 0.87 (0.87-0.88) |
| <60 years | 1.40 (1.35-1.46) | 1 (Reference) | 0.68 (0.64-0.74) | 0.83 (0.82-0.84) | 1.41 (1.35-1.47) | 1 (Reference) | 0.68 (0.64-0.72) | 0.83 (0.82-0.84) |
| P for interaction | 0.126 | | | | 0.199 | | | |
| **BMI** | | | | | | | | |
| Normal BMI | 1.32 (1.25-1.40) | 1 (Reference) | 0.85 (0.76-0.96) | 0.88 (0.86-0.90) | 1.32 (1.23-1.41) | 1 (Reference) | 0.80 (0.73-0.88) | 0.88 (0.86-0.90) |
| Abnormal BMI | 1.27 (1.24-1.30) | 1 (Reference) | 0.82 (0.77-0.87) | 0.88 (0.86-0.89) | 1.30 (1.27-1.34) | 1 (Reference) | 0.80 (0.77-0.84) | 0.88 (0.87-0.89) |
| P for interaction | 0.314 | | | | 0.306 | | | |
| **Comorbidities** | | | | | | | | |
| No | 1.24 (1.19-1.30) | 1 (Reference) | 0.89 (0.81-0.98) | 0.88 (0.87-0.89) | 1.30 (1.23-1.37) | 1 (Reference) | 0.88 (0.82-0.95) | 0.89 (0.88-0.91) |
| Yes | 1.23 (1.20-1.26) | 1 (Reference) | 0.84 (0.79-0.90) | 0.90 (0.88-0.91) | 1.24 (1.20-1.27) | 1 (Reference) | 0.80 (0.76-0.84) | 0.89 (0.89-0.91) |
| P for interaction | 0.161 | | | | 0.333 | | | |

This analysis adjusted for age, sex, racial background, educational level, occupational status, Townsend deprivation index, body mass index, healthy lifestyle score, comorbidities (chronic respiratory disease, chronic liver disease, hypertension, diabetes, and dyslipidemia), and laboratory measurements (eGFR_cr_, INFLA score, serum albumin, HDL-C, LDL-C, triglycerides, and UACR). Abbreviation: eGFR_diff_, the difference between cystatin C–based estimated glomerular filtration rate and creatinine-based estimated glomerular filtration rate; HR, hazard ratio; CI, confidence interval; INFLA score, Low-grade chronic inflammation score; HDL-C, high-density lipoprotein cholesterol; LDL-C, low-density lipoprotein cholesterol; UACR, Urinary albumin-creatinine ratio; CVD, Cardiovascular disease.

**Table S16.** Subgroup Analysis of The Association Between Cardiovascular Disease Mortality and eGFR_diff_

| **Characteristics** | **Non-Race-Based eGFR_diff_ (CVD mortality)** | | | | **Race-Related eGFR_diff_ (CVD mortality)** | | | |
| --- | --- | --- | --- | --- | --- | --- | --- | --- |
|  | **Negative <-15** | **Midrange -15 to 15** | **Positive ≥ 15** | **Per 10mL/min/1.73m2 increase** | **Negative <-15** | **Midrange -15 to 15** | **Positive ≥ 15** | **Per 10mL/min/1.73m2 increase** |
| **Sex** | | | | | | | | |
| Male | 1.45 (1.34-1.57) | 1 (Reference) | 0.69 (0.54-0.88) | 0.83 (0.80-0.86) | 1.47 (1.34-1.60) | 1 (Reference) | 0.69 (0.57-0.82) | 0.83 (0.80-0.86) |
| Female | 1.49 (1.33-1.67) | 1 (Reference) | 0.62 (0.44-0.87) | 0.77 (0.73-0.80) | 1.60 (1.41-1.80) | 1 (Reference) | 0.63 (0.48-0.83) | 0.77 (0.73-0.80) |
| P for interaction | 0.494 | | | | 0.384 | | | |
| **Age** | | | | | | | | |
| ≥60 years | 1.54 (1.43-1.66) | 1 (Reference) | 0.47 (0.35-0.62) | 0.79 (0.76-0.81) | 1.55 (1.42-1.68) | 1 (Reference) | 0.57 (0.47-0.69) | 0.79 (0.76-0.81) |
| <60 years | 1.79 (1.57-2.04) | 1 (Reference) | 0.69 (0.51-0.91) | 0.74 (0.71-0.78) | 1.93 (1.68-2.22) | 1 (Reference) | 0.59 (0.46-0.75) | 0.74 (0.71-0.78) |
| P for interaction | 0.021 | | | | 0.395 | | | |
| **BMI** | | | | | | | | |
| Normal BMI | 1.59 (1.34-1.87) | 1 (Reference) | 0.78 (0.51-1.17) | 0.80 (0.75-0.85) | 1.65 (1.36-1.99) | 1 (Reference) | 0.70 (0.50-0.98) | 0.80 (0.75-0.85) |
| Abnormal BMI | 1.51 (1.41-1.62) | 1 (Reference) | 0.61 (0.48-0.77) | 0.80 (0.77-0.82) | 1.57 (1.45-1.69) | 1 (Reference) | 0.64 (0.54-0.76) | 0.80 (0.77-0.82) |
| P for interaction | 0.394 | | | | 0.102 | | | |
| **Comorbidities** | | | | | | | | |
| No | 1.57 (1.36-1.81) | 1 (Reference) | 0.73 (0.49-1.07) | 0.78 (0.74-0.83) | 1.74 (1.47-2.05) | 1 (Reference) | 0.63 (0.46-0.85) | 0.78 (0.74-0.83) |
| Yes | 1.45 (1.35-1.56) | 1 (Reference) | 0.66 (0.52-0.84) | 0.82 (0.79-0.84) | 1.48 (1.37-1.60) | 1 (Reference) | 0.70 (0.59-0.83) | 0.82 (0.80-0.84) |
| P for interaction | 0.183 | | | | 0.195 | | | |

This analysis adjusted for age, sex, racial background, educational level, occupational status, Townsend deprivation index, body mass index, healthy lifestyle score, comorbidities (chronic respiratory disease, chronic liver disease, hypertension, diabetes, and dyslipidemia), and laboratory measurements (eGFR_cr_, INFLA score, serum albumin, HDL-C, LDL-C, triglycerides, and UACR). Abbreviation: eGFR_diff_, the difference between cystatin C–based estimated glomerular filtration rate and creatinine-based estimated glomerular filtration rate; HR, hazard ratio; CI, confidence interval; INFLA score, Low-grade chronic inflammation score; HDL-C, high-density lipoprotein cholesterol; LDL-C, low-density lipoprotein cholesterol; UACR, Urinary albumin-creatinine ratio; CVD, Cardiovascular disease.

**Table S17.** Subgroup Analysis of The Association Between All-cause Mortality and eGFR_diff_

| **Characteristics** | **Non-Race-Based eGFR_diff_ (All cause mortality)** | | | | **Race-Related eGFR_diff_ (All cause mortality)** | | | |
| --- | --- | --- | --- | --- | --- | --- | --- | --- |
|  | **Negative <-15** | **Midrange -15 to 15** | **Positive ≥ 15** | **Per 10mL/min/1.73m2 increase** | **Negative <-15** | **Midrange -15 to 15** | **Positive ≥ 15** | **Per 10mL/min/1.73m2 increase** |
| **Sex** | | | | | | | | |
| Male | 1.41 (1.35-1.47) | 1 (Reference) | 0.75 (0.66-0.85) | 0.85 (0.83-0.86) | 1.44 (1.38-1.51) | 1 (Reference) | 0.79 (0.72-0.86) | 0.85 (0.83-0.86) |
| Female | 1.42 (1.35-1.49) | 1 (Reference) | 0.77 (0.68-0.88) | 0.83 (0.81-0.84) | 1.48 (1.40-1.56) | 1 (Reference) | 0.76 (0.69-0.85) | 0.83 (0.81-0.84) |
| P for interaction | 0.146 | | | | 0.242 | | | |
| **Age** | | | | | | | | |
| ≥60 years | 1.46 (1.41-1.52) | 1 (Reference) | 0.67 (0.60-0.76) | 0.82 (0.81-0.83) | 1.49 (1.43-1.55) | 1 (Reference) | 0.71 (0.65-0.77) | 0.82 (0.81-0.83) |
| <60 years | 1.76 (1.66-1.87) | 1 (Reference) | 0.58 (0.50-0.66) | 0.76 (0.74-0.78) | 1.85 (1.73-1.97) | 1 (Reference) | 0.60 (0.54-0.67） | 0.76 (0.74-0.78) |
| P for interaction | 0.685 | | | | 0.925 | | | |
| **BMI** | | | | | | | | |
| Normal BMI | 1.60 (1.49-1.73) | 1 (Reference) | 0.80 (0.67-0.96) | 0.80 (0.78-0.83) | 1.69 (1.55-1.84) | 1 (Reference) | 0.79 (0.69-0.91) | 0.80 (0.78-0.83) |
| Abnormal BMI | 1.39 (1.34-1.44) | 1 (Reference) | 0.75 (0.68-0.83) | 0.84 (0.83-0.86) | 1.44 (1.38-1.50) | 1 (Reference) | 0.78 (0.72-0.84) | 0.84 (0.83-0.86) |
| P for interaction | 0.811 | | | | 0.745 | | | |
| **Comorbidities** | | | | | | | | |
| No | 1.40 (1.32-1.50) | 1 (Reference) | 0.85 (0.73-0.98) | 0.86 (0.84-0.88) | 1.52 (1.41-1.64) | 1 (Reference) | 0.88 (0.79-0.99) | 0.86 (0.84-0.88) |
| Yes | 1.42 (1.37-1.48) | 1 (Reference) | 0.75 (0.67-0.84) | 0.83 (0.82-0.84) | 1.45 (1.39-1.51) | 1 (Reference) | 0.75 (0.69-0.81) | 0.83 (0.82-0.84) |
| P for interaction | 0.933 | | | | 0.229 | | | |

This analysis adjusted for age, sex, racial background, educational level, occupational status, Townsend deprivation index, body mass index, healthy lifestyle score, comorbidities (chronic respiratory disease, chronic liver disease, hypertension, diabetes, and dyslipidemia), and laboratory measurements (eGFR_cr_, INFLA score, serum albumin, HDL-C, LDL-C, triglycerides, and UACR). Abbreviation: eGFR_diff_, the difference between cystatin C–based estimated glomerular filtration rate and creatinine-based estimated glomerular filtration rate; HR, hazard ratio; CI, confidence interval; INFLA score, Low-grade chronic inflammation score; HDL-C, high-density lipoprotein cholesterol; LDL-C, low-density lipoprotein cholesterol; UACR, Urinary albumin-creatinine ratio; CVD, Cardiovascular disease.

**Figure S1.** Flowchart of study participants


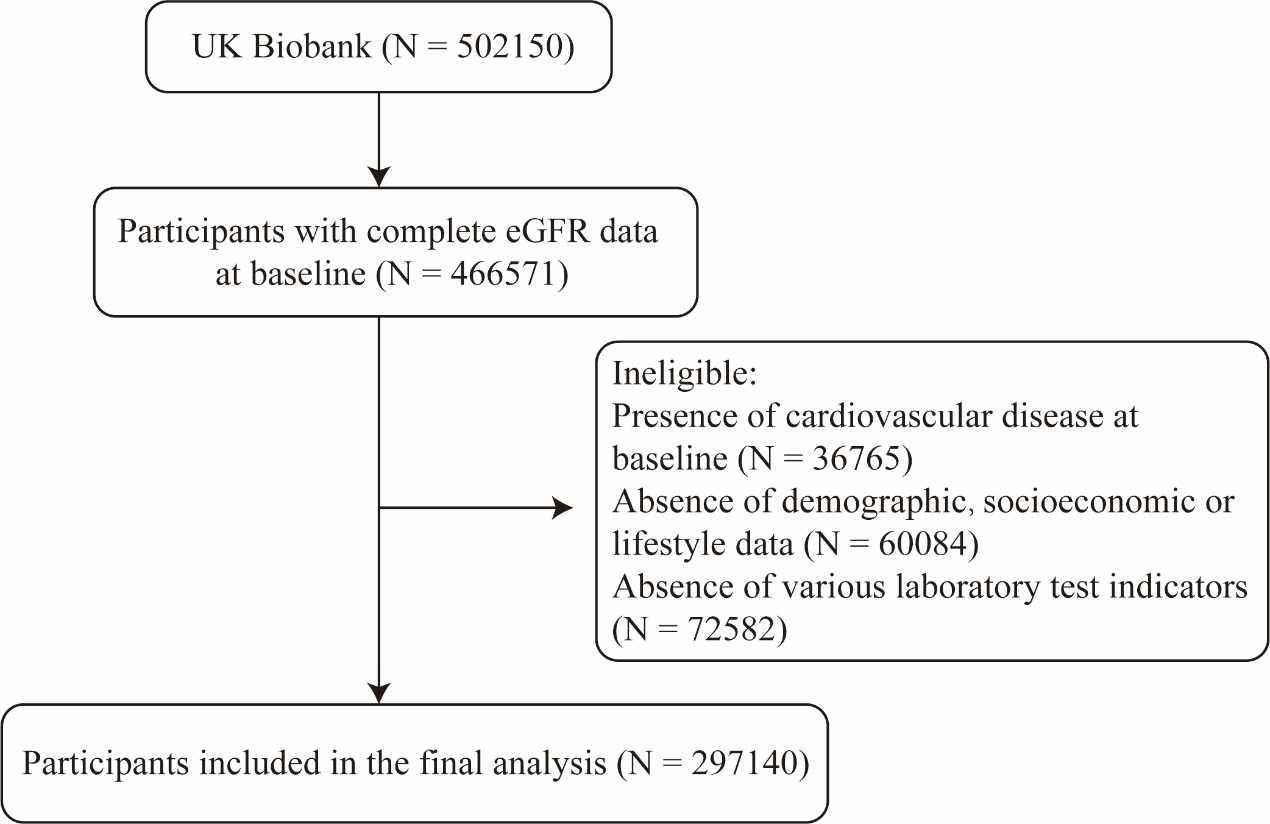


**Figure S2.** Restrictive Cubic Spine Plot of Cardiovascular-related Morbidity According to Non-Race-Based eGFR_diff_


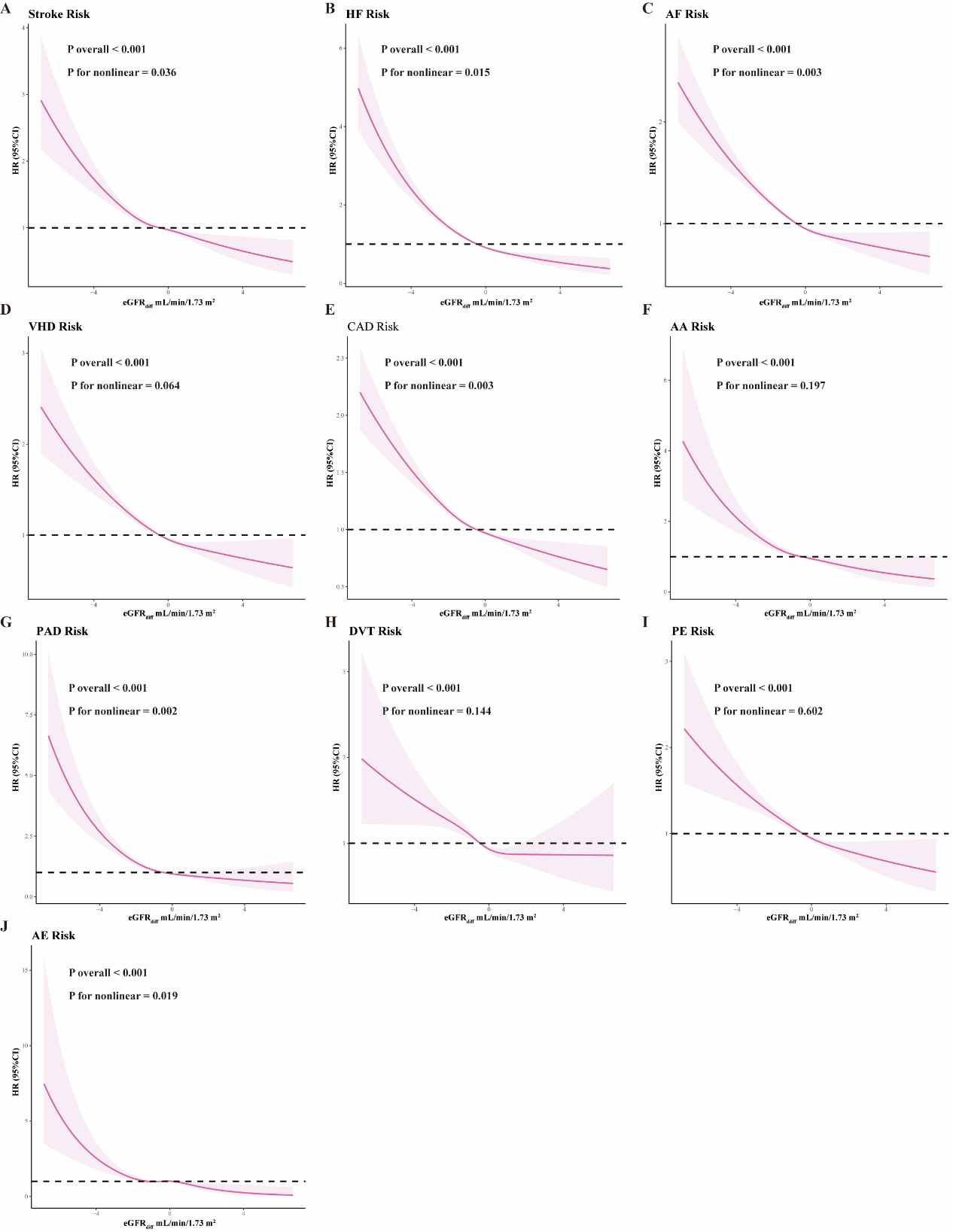


This analysis adjusted for age, sex, racial background, educational level, occupational status, Townsend deprivation index, body mass index, healthy lifestyle score, comorbidities (chronic respiratory disease, chronic liver disease, hypertension, diabetes, and dyslipidemia), and laboratory measurements (eGFR_cr_, INFLA score, serum albumin, HDL-C, LDL-C, triglycerides, and UACR). Abbreviation: eGFR_diff_, the difference between cystatin C–based estimated glomerular filtration rate and creatinine-based estimated glomerular filtration rate; HR, hazard ratio; CI, confidence interval; INFLA score, Low-grade chronic inflammation score; HDL-C, high-density lipoprotein cholesterol; LDL-C, low-density lipoprotein cholesterol; UACR, Urinary albumin-creatinine ratio; HF, Heart failure Heart failure; AF, Atrial fibrillation; VHD, Valvular heart disease; CAD, Coronary atherosclerotic heart disease; AA, Aortic aneurysm; PAD, Peripheral artery disease; DVT, Deep vein thrombosis; PE, Pulmonary embolism; AE, Arterial embolism.

**Figure S3.** Restrictive Cubic Spine Plot of Cardiovascular-related Morbidity and Mortality According to Race-Related eGFR_diff_


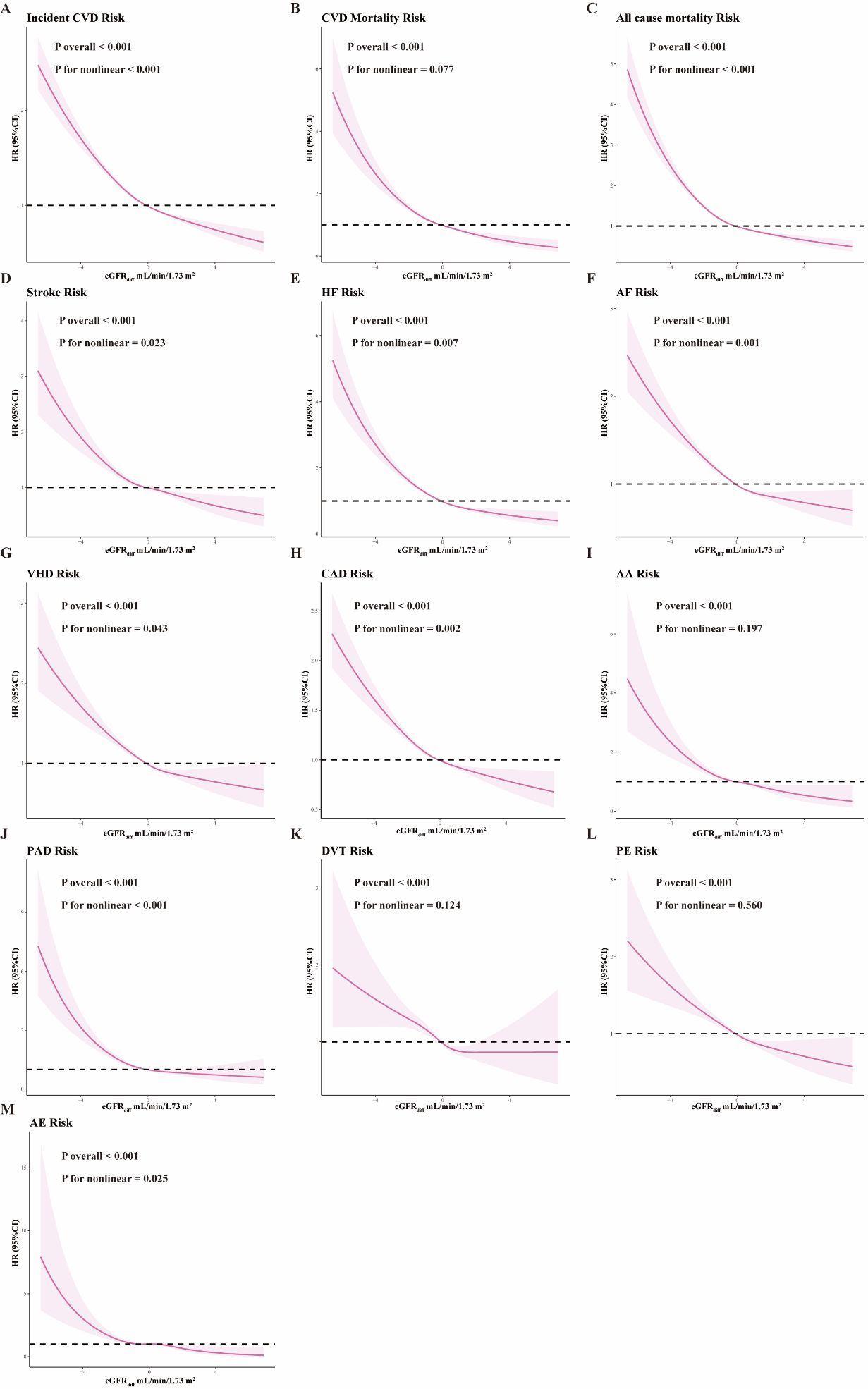


This analysis adjusted for age, sex, racial background, educational level, occupational status, Townsend deprivation index, body mass index, healthy lifestyle score, comorbidities (chronic respiratory disease, chronic liver disease, hypertension, diabetes, and dyslipidemia), and laboratory measurements (eGFR_cr_, INFLA score, serum albumin, HDL-C, LDL-C, triglycerides, and UACR). Abbreviation: eGFR_diff_, the difference between cystatin C–based estimated glomerular filtration rate and creatinine-based estimated glomerular filtration rate; HR, hazard ratio; CI, confidence interval; INFLA score, Low-grade chronic inflammation score; HDL-C, high-density lipoprotein cholesterol; LDL-C, low-density lipoprotein cholesterol; UACR, Urinary albumin-creatinine ratio; CVD, Cardiovascular disease; HF, Heart failure Heart failure; AF, Atrial fibrillation; VHD, Valvular heart disease; CAD, Coronary atherosclerotic heart disease; AA, Aortic aneurysm; PAD, Peripheral artery disease; DVT, Deep vein thrombosis; PE, Pulmonary embolism; AE, Arterial embolism.
